# DNA methylation in peripheral blood leukocytes for the association with glucose metabolism and invasive breast cancer

**DOI:** 10.1186/s13148-023-01435-7

**Published:** 2023-02-13

**Authors:** Su Yon Jung, Parveen Bhatti, Matteo Pellegrini

**Affiliations:** 1 Translational Sciences Section, School of Nursing, University of California, Los Angeles, 700 Tiverton Ave, 3-264 Factor Building, Los Angeles, CA 90095 USA; 2 Jonsson Comprehensive Cancer Center, University of California, Los Angeles, Los Angeles, CA 90095 USA; 3 Cancer Control Research, BC Cancer Research Institute, Vancouver, BC Canada; 4 School of Population and Public Health, University of British Columbia, Vancouver, BC Canada; 5 Department of Molecular, Cell and Developmental Biology, Life Sciences Division, University of California, Los Angeles, Los Angeles, CA 90095 USA

**Keywords:** Epigenetic signatures, DNA methylation, Glucose homeostasis, Obesity, Invasive breast cancer, Postmenopausal women

## Abstract

**Background:**

Insulin resistance (IR) is a well-established factor for breast cancer (BC) risk in postmenopausal women, but the interrelated molecular pathways on the methylome are not explicitly described. We conducted a population-level epigenome-wide association (EWA) study for DNA methylation (DNAm) probes that are associated with IR and prospectively correlated with BC development, both overall and in BC subtypes among postmenopausal women.

**Methods:**

We used data from Women’s Health Initiative (WHI) ancillary studies for our EWA analyses and evaluated the associations of site-specific DNAm across the genome with IR phenotypes by multiple regressions adjusting for age and leukocyte heterogeneities. For our analysis of the top 20 IR-CpGs with BC risk, we used the WHI and the Cancer Genomic Atlas (TCGA), using multiple Cox proportional hazards and logit regressions, respectively, accounting for age, diabetes, obesity, leukocyte heterogeneities, and tumor purity (for TCGA). We further conducted a Gene Set Enrichment Analysis.

**Results:**

We detected several EWA-CpGs in *TXNIP, CPT1A, PHGDH,* and *ABCG1.* In particular, cg19693031 in *TXNIP* was replicated in all IR phenotypes, measured by fasting levels of glucose, insulin, and homeostatic model assessment-IR. Of those replicated IR-genes, 3 genes (*CPT1A, PHGDH,* and *ABCG1*) were further correlated with BC risk; and 1 individual CpG (cg01676795 in *POR*) was commonly detected across the 2 cohorts.

**Conclusions:**

Our study contributes to better understanding of the interconnected molecular pathways on the methylome between IR and BC carcinogenesis and suggests potential use of DNAm markers in the peripheral blood cells as preventive targets to detect an at-risk group for IR and BC in postmenopausal women.

**Supplementary Information:**

The online version contains supplementary material available at 10.1186/s13148-023-01435-7.

## Background

Breast cancer (BC), the topmost leading cause of cancer incidence in women of the USA and worldwide [1, 2], is a heterogeneous disease with multiple clinical, histopathological, and molecular subtypes, which is characterized by both genetic and epigenetic alterations [3, 4]. For epigenetic events, DNA methylation (DNAm) is a well-characterized major epigenetic modification that involves mitotically heritable and reversible attachment of methyl groups at the 5′ carbon of cytosine in CpG dinucleotides (CpGs), influencing DNA transcription without altering the DNA sequence [5, 6]. Whereas several DNAm studies for BC initiation and progression support global hypomethylation [7–9] and focal hypermethylation, such that some tumor-suppressor genes are frequently hypermethylated at CpG islands and promoters, thus being inactivated [8, 10, 11], the role of the epigenetic mechanisms in BC tumorigenesis has not been conclusive. For example, there is no consistent trend toward an association between identified CpGs and the risk of BC across studies, suggesting the need for large population-level epigenetic studies, which prospectively evaluate BC development, specifically in BC molecular-subtype stratifications [7, 8].

In particular, among postmenopausal women, the obesity–insulin resistance (IR) connection is a well-established factor for BC risk/progression [1, 12–15], but their interrelated molecular pathways on the methylome have not been established. In detail, IR and type 2 diabetes (T2DM) are influenced by environmental and genomic factors as well as by their interplay [16–19]. In prior genome-wide association studies (GWASs), a majority of genes are associated with insulin secretion, pointing to pancreatic islet defects but does not represent impaired insulin action [19–21]; and these genes explain a small portion of the estimated heritability [19, 22]. The analysis of epigenetics may address these issues. For example, obesity status/adipose tissues and long-term exposure of beta cell lines to hyperglycemia altered DNAm of genes involved in glucose metabolism and their gene expression, leading to impaired insulin secretion as well as sensitivity [17, 23–30]. Thus, aberrant DNAm may directly influence the function of pancreatic beta cells as well as other organs involved in glucose homeostasis. Also, considering that age, as measured via epigenetic age [31, 32], influences DNAm, changes in DNAm that are associated with IR account for aging in the methylome and thus may be a better indicator of inter-/intra-individual genomic variability of IR. As such, DNAm can be a biomarker of decreased insulin sensitivity, but few epigenome-wide association studies (EWASs) have so far examined DNAm in IR [30, 33–35]. In addition, the previous EWASs for obesity/metabolic syndrome showed limited evidence owing to a lack of findings’ validation and no comparison of findings between peripheral blood and tissues.

Further, one study [32] for IR in T2DM pancreatic islets reported that differentially methylated genes were enriched in pathways of cancer and *MAPK* signaling, suggesting a close link of epigenomic mechanisms between IR and cancer. Given that even a modest change in DNAm causes a substantial effect on gene expression and that in late-onset disease, it has a large effect on disease over a long time period [36], DNAm markers can serve as a biomarker to detect an early at-risk group for morbid conditions, such as IR and BC, even several years before the clinical diagnosis is made.

Our study was a population-level EWAS to detect DNAm probes that are associated with IR phenotypes and that, by using data from a prospective evaluation of BC development, are further directly correlated with BC risk, both overall and in BC subtypes among postmenopausal women. DNAm is tissue specific, but the correlations between peripheral blood and tissue are gene specific. For example, the methylation levels of several genes in relation to IR, T2DM, and/or BC are highly correlated between peripheral blood and tissue [37–40]. Thus, we first conducted a peripheral blood leukocytes (PBLs)–based EWAS and compared the methylation levels of detected CpGs with those of the CpGs within BC and adjacent normal breast tissues. Corresponding to the results in a published study [41] of gene-methylation parallelisms between peripheral blood cells and tissues in glucose metabolism, the PBLs may be the best non-invasive alternative tissue, standing for a surrogate DNAm marker that reflects multiple glucometabolic pathways. With the detected EWA-based IR-CpGs, we further tested for the associations with BC risk in PBLs and conducted validation tests in BC tissues. This allowed us to determine whether our IR-CpGs at genome-wide significance that were associated with BC risk are systemic or tissue specific or common in both.

## Materials and methods

### Study population

**Fig. 1:**
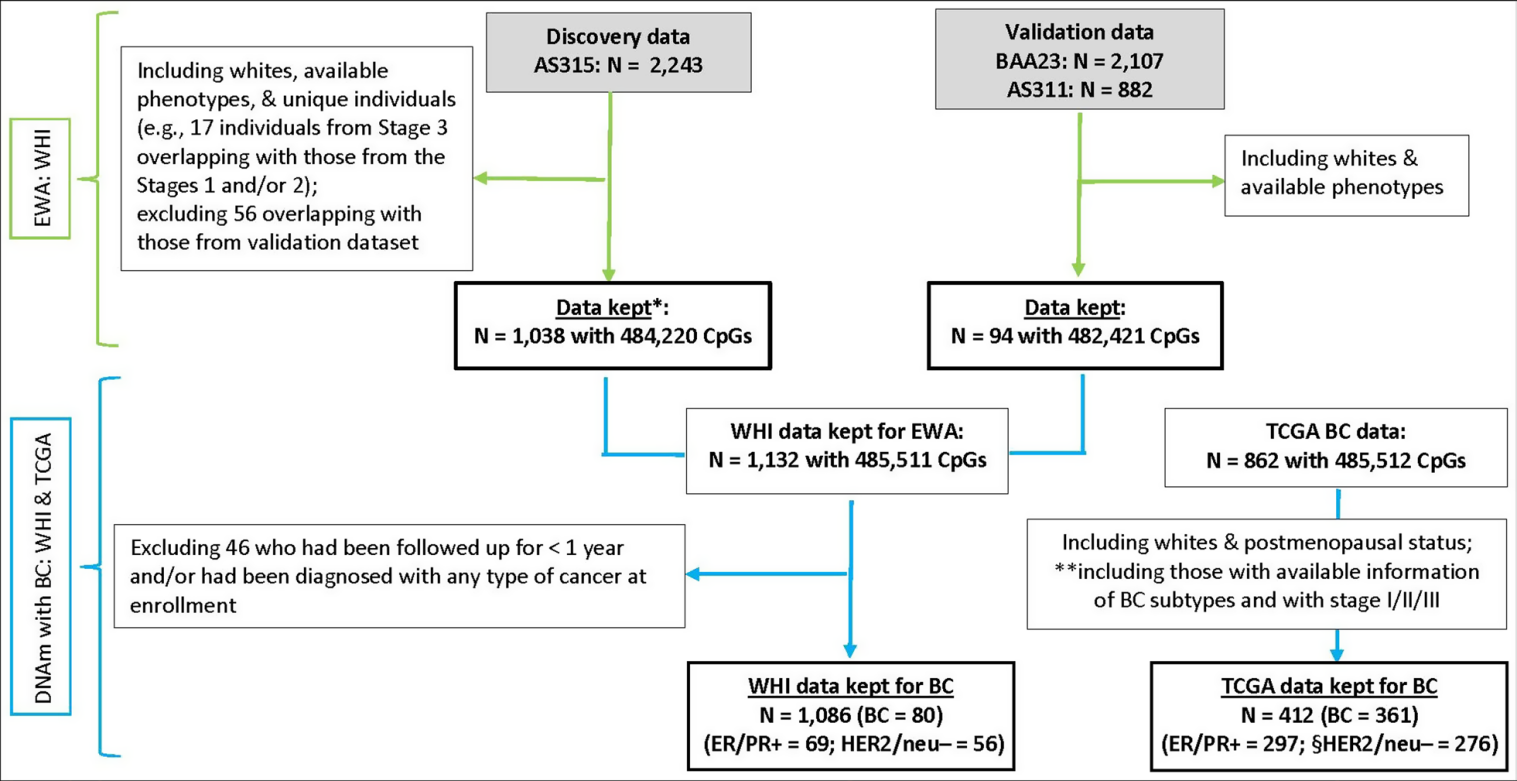
Diagram of EWA and BC study populations from the WHI and TCGA cohorts. *BC* Breast cancer, *CpGs* CpG dinucleotide, *DNAm* DNA methylation, *ER/PR* + Estrogen receptor/progesterone receptor–positive, *EWA* Epigenome-wide association, *HER2/neu–* Human epidermal growth factor receptor-2–negative; *TCGA* The Cancer Genomic Atlas, *WHI* Women’s Health Initiative. * Individuals within Stage 2 had DNAm data measured at 2 visits and, for the analysis, the DNAm of those with a shorter interval between enrollment and blood draw were selected. ** Those selection criteria were applied to TCGA BC tissues. § The cases of HER2/neu– contained 49 (13% of BC cases) triple negatives

Our EWA analysis used data from the Women’s Health Initiative (WHI) cohort, a large, prospective study of postmenopausal women, whose ages were 50–79 years at the time of enrollment between 1993 and 1998 at 40 clinical centers in the USA, consisting of 2 study arms, namely the clinical trial (CT) and the observational study (OS) [42]. For DNAm data, we included 3 WHI ancillary studies (ASs) with available genome-wide DNAm measured in PBLs (Fig. 1): for discovery, AS315 (Epigenetic Mechanisms of Particulate Matter-Mediated Cardiovascular Disease, random minority oversample from WHI CT, *n* = 2,243); for validation, Broad Agency Award (BAA23, Integrative Genomics for Risk of Coronary Heart Disease [CHD] and Related Phenotypes, case–control study of CHD from WHI CT and OS, *n* = 2,107) combined with AS311 (Bladder Cancer and Leukocyte Methylation, matched case–control study of bladder cancer from WHI CT and OS, *n* = 882) [43, 44]. Racial/ethnic variation exists in BC-related DNAm [37, 45]; for the purpose of our EWA analysis, we restricted the study population to those women who reported their race or ethnicity as non-Hispanic white, a majority of the WHI ASs population, and who had available IR phenotypes assessed via fasting blood levels of glucose (FG) and insulin (FI) (*n* = 1,132).

For our analysis of the validated IR-CpGs with the risk of BC development, our discovery cohort included those 3 WHI ASs with available BC outcomes but excluding women (*n* = 46) who had been followed up for less than 1 year and/or had been diagnosed with any type of cancer at enrollment, leaving a total of 1,086 women (Fig. 1). These women had been followed up through March 6, 2021, with a mean of 17 years follow-up, and 80 of them had developed invasive BC. Our replication cohort was derived from the Cancer Genomic Atlas (TCGA) BC Study (*n* = 862), housing tissue-derived genome-wide DNAm data and molecular profiles of different BC subtypes from BC tissues [46]. Our analyses for BC were restricted to women who are white and postmenopausal with available BC subtypes, but distant-metastasis free, resulting in a total of 412 (= 361 BC tissues + 51 adjacent normal breast tissues) (Fig. 1). The institutional review boards of each WHI clinical center and the University of California, Los Angeles, approved this study.

### Data collection and BC outcome

Participants enrolled in the WHI completed self-administered questionnaires at screening, providing demographic information (e.g., age, race) and medical histories, such as DM. Trained staff obtained anthropometric measurements, including height, weight, and waist and hip circumferences at baseline. Invasive BC development was initially ascertained through self-report of a new cancer diagnosis by all participants, further determined by a committee of physicians on the basis of a review of the patients’ medical records and pathology and cytology reports, and coded into the central WHI database according to the National Cancer Institute’s Surveillance, Epidemiology, and End-Results guidelines [47]. The time from enrollment until BC development, censoring, or study end-point was measured as the number of days and then converted into years.

BC patient data from TCGA used in this study include information on age, race, menopausal status, and diagnosed tumor subtype and stage. For the study purpose, data from primary invasive BC tissues and normal breast tissues adjacent to BC (either primary or metastatic) tissues were analyzed.

### Epigenome-wide DNAm array and laboratory methods

Using peripheral blood leukocytes isolated from the fasting blood of the WHI participants, we extracted DNA and measured DNAm via the Illumina 450 BeadChip (Illumina Inc.; San Diego, CA) at up to 485,511 CpG sites. DNAm levels (β values) were calculated as the ratio of intensities between the methylated and unmethylated probes, ranging from 0 (completely unmethylated) to 1 (completely methylated) [48]. DNAm was beta-mixture quantile (BMIQ)-normalized, [49] and batched-adjusted for stage and plate by using the empirical Bayes methods [50] or by using random intercept for plate and chip and a fixed effect for row. Leukocyte heterogeneities were estimated to be adjusted for in the analysis using Houseman’s method [51] (for CD4^+^ T cell, natural killer cell, monocyte, and granulocyte) and Hovarth’s method [52] (for plasma blast, CD8^+^CD28^–^CD45RA^–^ T cell, and naïve CD8 T cell).

In TCGA, tissue-derived genome-wide DNAm was analyzed by using the Illumina Infinium450K array and, using minfi v.1.42.0, was normalized via normal-exponential out-of-band (Noob) background correction [53]. The tumor purity and cell-type proportions (cancer and normal epithelial, stromal, and immune cells) of each tumor sample were estimated by using the R InfiniumPurify v. 1.3.1 [54] and RefFreeEWAS V.2.2 [55], respectively.

Serum samples from the WHI participants fasting at least 8 h were drawn at enrollment by trained phlebotomists and assayed for glucose and insulin concentrations using the hexokinase method on a Hitachi 747 analyzer (Boehringer Mannheim Diagnostics, Indianapolis, IN) for glucose, and by radioimmunoassay (Linco Research, Inc., St. Louis, MO) or automated ES300 method (Boehringer Mannheim Diagnostics, Indianapolis, IN) for insulin. Results from the 2 methods for insulin measurement were comparable at insulin concentrations < 60 μIU/ml, and the intra-class correlation coefficient with repeatedly measured insulin was 0.7 [56]. Homeostatic model assessment–IR (HOMA-IR), as a surrogate of IR, was estimated as glucose (unit: mg/dl) × insulin (unit: μIU/ml) / 405 [57].

### Statistical analysis

For the DNAm site-specific analysis across the genome with IR phenotypes, each phenotype was log-transformed as a result of tests conducted for linear assumption and normality distribution and was also categorized as follows: FG, FI, and HOMA-IR, using 100 mg/dl, 8.6μIU/ml, and 3.0 (respectively), corresponding to the cut points of the American Heart Association/National Heart, Lung, and Blood Institute, the International Diabetes Federation, and the Adult Treatment Panel III for metabolic syndrome [58, 59]. The association between DNAm and each phenotype was evaluated via multiple linear and logistic regressions, adjusting for age and leukocyte heterogeneities. The summary of the leukocyte proportions is provided in Additional file 1: Table S1. A 2-sided *p* < 1E–007 (discovery) and 0.05 / number of the discovered CpGs (replication), providing Bonferroni correction, were considered statistically significant. Results were combined across discovery and replication in a meta-analysis assuming a fixed–effect model.

With the selected top 20 CpG sites that were most statistically significant after multiple-comparison corrections, we next performed in the WHI data the multiple Cox proportional hazards regression for BC development overall and within BC subtypes, with an assumption test via a Schoenfeld residual plot and rho, by accounting for age, having ever been treated for diabetes, body mass index (BMI), waist-to-hip ratio (WHR), and leukocyte heterogeneities. Using TCGA data, we further conducted validation tests of the top 20 CpGs with BC risk by using logit regression that was adjusted for age, tumor purity, and cell-type composition both overall and in the BC subtypes. For the analysis of BC risk, the modeled CpGs in both cohorts were further standardized across samples; thus, the effect size reflected a 1 standardized deviation increase in DNAm on BC risk. Given that this testing was performed on the basis of our hypothesis-driven questions (i.e., IR-DNAm in association with BC systemically or in tissues), a 2-tailed *p* < 0.05 was considered significant.

Differences in methylation levels of the modeled CpGs by IR phenotypes in the PBLs and by BC risk in each of PBLs and tissues, as well as differences in the DNAm status between the PBLs and tissues among women with BC and those without BC, were tested via unpaired 2-sample t tests. If β values were skewed or had outliers, Mann–Whitney/Wilcoxon’s rank-sum test was used. With the CpGs at genome-wide significance in the discovery and those of which were associated with BC risk in either TCGA or WHI, we finally conducted a Gene Set Enrichment Analysis (GSEA) by IR phenotypes and by BC subtypes, respectively, using the WebGestalt [60]. All statistical analyses were performed using R.

## Results

### Epigenome-wide association of DNAm and IR phenotypes.

**Fig. 2:**
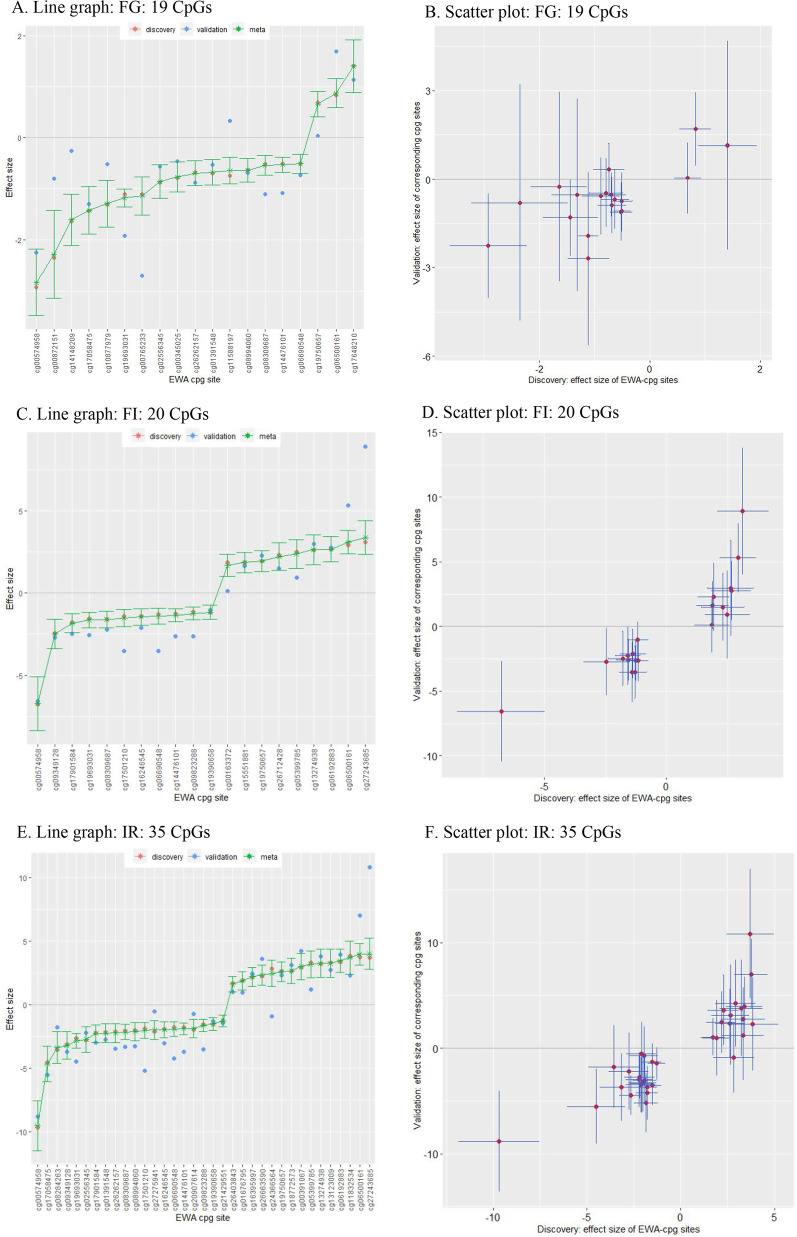
Comparison among the effect sizes of EWA-CpGs in FG, FI, and HOMA-IR as continuous variable in discovery, validation, and meta-analyses. (*CpG* CpG dinucleotide, *EWA* Epigenome-wide association, *FG and FI* Fasting levels of glucose and insulin, *HOMA-IR* homeostatic model assessment-insulin resistance). **A** Line graph: FG: 19 CpGs; **B** Scatter plot: FG: 19 CpGs; **C** Line graph: FI: 20 CpGs; **D** Scatter plot: FI: 20 CpGs; **E** Line graph: IR: 35 CpGs; **F** Scatter plot: IR: 35 CpGs

Among 484,220 CpGs in the discovery data, we found several differentially methylated CpGs associated with each IR phenotype (FG, FI, and HOMA-IR) and further validated them. In detail, 19 CpGs were associated with FG, the level of which was analyzed as a continuous variable; of those, 1 CpG (cg19693031 in *TXNIP*) was further validated, with *p* < 2.6E–03(= 0.05/19) (Table 1, Figs. 2A and B). This same CpG was also replicated in the analysis for FG as a categorical variable, showing the same direction as the effect size estimated in the FG analysis as a continuous variable (Additional file 1: Table S2). Of 20 CpGs in relation to FI as a continuous variable in discovery, 7 CpGs were further validated, with *p* < 2.5E–03 (= 0.05/20; Table 2, Figs. 2C and D). Of those 7 CpGs, 1 CpG (cg00574957 in *CPT1A*) was also replicated in the analysis of FI as a categorical variable (Additional file 1: Table S3); in both linear and logistic analyses, this CpG was negatively associated with FI. For HOMA-IR as a continuous variable, 35 CpGs were detected in discovery; 7 of those were further validated (*p* < 1.4E–03; Table 3, Figs. 2E and F). In the analysis of HOMA-IR as a categorical variable, 4 of the validated 7 CpGs (cg14476101 in *PHGDH*, cg19693031 in *TXNIP*, cg00574958 in *CPT1A*, and cg06500161 in *ABCG1*) were also detected in discovery, yielding the same directions as those of effect sizes estimated in the linear analyses, but none of them were further validated (Additional file 1: Table S4). Finally, we conducted a meta-analysis of all the detected epigenome-wide CpGs by combining their discovery and replication data. We detected 1 CpG (cg19693031 in *TXNIP*) that was replicated inversely related to FG, FI, and HOMA-IR each as a continuous variable; and that CpG was also significant at the epigenome-wide level in association with FG and HOMA-IR each as a categorical variable.

**Table 1:** Genome-wide scan of DNA methylation for an association with fasting glucose concentrations (as a continuous variable)

Chr	CpG site§	Position	Discovery	Validation		Meta − analysis			CpG context	Gene	Gene region
			Effect†a (95% CI)	Effect a (95% CI)	*p*	Effect (95% CI)	*p*	*p*_Q_¥			
chr1	cg14476101	120,255,992	** − 0.50 (− 0.66, − 0.35)**	** − **1.08 (**− **1.79, − 0.38)	0.003	** − 0.53 (− 0.68, − 0.38)**	**4.37E − 12**	0.111	S Shore	*PHGDH*	Body
chr1	cg19693031	145,441,552	** − 1.11 (− 1.30, − 0.93)**	** − 1.93 (− 2.55, − 1.30)**	** < 0.0026**	** − 1.18 (− 1.36, − 1.00)**	**1.10E − 38**	0.013	OpenSea	*TXNIP*	3'UTR
chr2	cg00765233*	235,877,260	** − 1.11 (− 1.49, − 0.74)**	** − **2.70 (**− **5.67, 0.28)	0.075	** − 1.14 (− 1.51, − 0.77)**	**1.92E − 09**	0.294	OpenSea	*SH3BP4*	5'UTR
chr4	cg06690548	139,162,808	** − 0.50 (− 0.70, − 0.31)**	** − **0.74 (− 1.70, 0.23)	0.132	** − 0.51 (− 0.70, − 0.32)**	**9.65E − 08**	0.638	OpenSea	*SLC7A11*	Body
chr8	cg00345025	743,875	** − 0.79 (− 1.09, − 0.49)**	− 0.46 (− 1.65, 0.73)	0.444	** − 0.77 (− 1.06, − 0.48)**	**2.35E − 07**	0.594	N Shore		Intergenic
chr9	cg14148209*	7,543,165	** − 1.65 (− 2.16, − 1.13)**	− 0.25 (− 3.52, 3.01)	0.877	** − 1.61 (− 2.12, − 1.11)**	**3.93E − 10**	0.402	OpenSea		Intergenic
chr10	cg01391548*	97,068,696	** − 0.70 (− 0.96, − 0.44)**	− 0.53 (− 1.28, 0.23)	0.167	** − 0.68 (− 0.92, − 0.43)**	**6.21E − 08**	0.675	OpenSea		Intergenic
chr10	cg02556345	124,181,965	** − 0.88 (− 1.22, − 0.54)**	− 0.57 (− 1.89, 0.76)	0.398	** − 0.86 (− 1.19, − 0.53)**	**2.60E − 07**	0.648	OpenSea	*PLEKHA1*	Body
chr10	cg08994060	6,214,026	** − 0.64 (− 0.88, − 0.39)**	− 0.69 (− 1.71, 0.32)	0.179	** − 0.64 (− 0.87, − 0.40)**	**1.02E − 07**	0.915	OpenSea	*PFKFB3*	Body
chr10	cg26262157	6,214,079	** − 0.68 (− 0.94, − 0.43)**	− 0.89 (− 1.85, 0.08)	0.070	** − 0.70 (− 0.94, − 0.45)**	**2.09E − 08**	0.685	OpenSea	*PFKFB3*	Body
chr11	cg00574958	68,607,622	** − 2.92 (− 3.62, − 2.23)**	− 2.26 (− 4.05, − 0.46)	0.014	** − 2.83 (− 3.48, − 2.19)**	**7.60E − 18**	0.494	N Shore	*CPT1A*	5'UTR
chr11	cg11588197	128,391,494	** − 0.74 (− 1.02, − 0.47)**	0.33 (− 0.56, 1.22)	0.462	** − 0.65 (− 0.91, − 0.38)**	**1.34E − 06**	0.022	N Shore	*ETS1*	Body
chr11	cg17058475	68,607,737	** − 1.44 (− 1.94, − 0.95)**	− 1.30 (− 2.63, 0.02)	0.054	** − 1.42 (− 1.89, − 0.96)**	**1.48E − 09**	0.845	N Shore	*CPT1A*	5'UTR
chr12	cg10877979	131,569,407	** − 1.31 (− 1.77, − 0.85)**	− 0.52 (− 3.81, 2.76)	0.753	** − 1.29 (− 1.75, − 0.84)**	**3.03E − 08**	0.637	N Shelf	*GPR133*	Body
chr13	cg19750657	38,935,967	**0.69 (0.44, 0.94)**	0.04 (− 1.17, 1.24)	0.951	**0.66 (0.41, 0.91)**	**1.53E − 07**	0.293	OpenSea	*UFM1*	3'UTR
chr20	cg00872151	59,964,387	** − 2.36 (− 3.23, − 1.48)**	− 0.80 (− 4.86, 3.26)	0.697	** − 2.29 (− 3.14, − 1.43)**	**1.62E − 07**	0.456	N Shore	*CDH4*	Body
chr21	cg06500161*	43,656,587	**0.83 (0.53, 1.12)**	1.69 (0.43, 2.94)	0.009	**0.87 (0.59, 1.15)**	**1.68E − 09**	0.184	S Shore	*ABCG1*	Body
chr21	cg08309687**	35,320,596	** − 0.52 (− 0.71, − 0.32)**	− 1.11 (− 2.12, − 0.10)	0.032	** − 0.54 (− 0.73, − 0.35)**	**3.42E − 08**	0.254	OpenSea		Intergenic
chr21	cg17648210	46,352,817	**1.41 (0.88, 1.93)**	1.14 (− 2.45, 4.73)	0.530	**1.40 (0.88, 1.92)**	**1.16E − 07**	0.883	Island		Intergenic

*Chr* Chromosome, *CI* Confidence interval, *CpG* CpG dinucleotide, *Effect* Effect size; *UTR* Untranslated region. Numbers in bold face are statistically significant

^§^ Annotation used R v.0.6.0.*IlluminaHumanMethylation450kanno.ilmn12.hg19: Annotation for Illumina's 450 k methylation arrays*

^†^ Each effect size of the CpGs in the discovery stage was at the epigenome-wide significance level (*p* < 1E-007)

^a^ Effect size adjusted by age and leukocyte heterogeneities (CD8^+^CD28^−^CD45RA^−^ T cell, naïve CD8 T cell, plasma blast, CD4^+^ T cell, natural killer cell, monocyte, and granulocyte)

¥ *p* value for Cochran’s Q

^*^ Enhancer associated

^**^ Enhancer and promoter associated

**Table 2:** Genome-wide scan of DNA methylation for an association with fasting insulin concentrations (as a continuous variable)

Chr	CpG site§	Position	Discovery	Validation		Meta-analysis			CpG context	Gene	Gene region
			Effect† (95% CI)	Effect (95% CI)	*p*	Effect (95% CI)	*p*	*p*_Q_¥			
chr1	cg05399785	3,564,031	**2.49 (1.58, 3.40)**	0.93 (**− **2.51, 4.36)	0.592	**2.38 (1.50, 3.27)**	**1.17E-07**	0.383	N Shelf	*WDR8*	Body
chr1	cg09823288	239,552,128	**− 1.15 (− 1.56, − 0.74)**	**− 2.62 (− 4.21, − 1.03)**	**0.001**	**− 1.24 (− 1.64, − 0.85)**	**8.92E-10**	0.074	S Shore		Intergenic
chr1	cg14476101	120,255,992	**− 1.27 (− 1.67, − 0.87)**	**− 2.65 (− 4.22, − 1.07)**	**0.001**	**− 1.35 (− 1.74, − 0.97)**	**4.55E-12**	0.093	S Shore	*PHGDH*	Body
chr1	cg16246545	120,255,941	**− 1.39 (− 1.92, − 0.87)**	**− **2.13 (**− **4.06, **− **0.20)	0.031	**− 1.45 (− 1.95, − 0.94)**	**2.01E-08**	0.466	S Shore	*PHGDH*	Body
chr1	cg17901584*	55,353,706	**− 1.79 (− 2.39, − 1.19)**	**− **2.47 (**− **4.60, **− **0.35)	0.023	**− 1.84 (− 2.42, − 1.27)**	**3.75E-10**	0.541	S Shore	*DHCR24*	TSS1500
chr1	cg19693031	145,441,552	**− 1.55 (− 2.05, − 1.05)**	**− 2.57 (− 4.17, − 0.96)**	**0.002**	**− 1.64 (− 2.12, − 1.17)**	**1.51E-11**	0.230	OpenSea	*TXNIP*	3'UTR
chr4	cg06690548	139,162,808	**− 1.30 (− 1.79, − 0.80)**	**− 3.51 (− 5.58, − 1.44)**	**0.001**	**− 1.42 (− 1.90, − 0.94)**	**7.80E-09**	0.038	OpenSea	*SLC7A11*	Body
chr6	cg17501210	166,970,252	**− 1.41 (− 1.93, − 0.89)**	**− **3.53 (**− **5.87, **− **1.19)	0.004	**− 1.51 (− 2.02, − 1.00)**	**5.67E-09**	0.079	OpenSea	*RPS6KA2*	Body
chr7	cg19390658*	30,636,176	**− 1.18 (− 1.62, − 0.74)**	**− **1.03 (**− **2.43, 0.38)	0.150	**− 1.17 (− 1.59, − 0.74)**	**6.22E-08**	0.836	S Shore	*GARS*	Body
chr8	cg00163372	128,752,988	**1.86 (1.15, 2.58)**	0.11 (**− **1.98, 2.21)	0.915	**1.67 (1.00, 2.35)**	**1.18E-06**	0.116	S Shore	*MYC*	Body
chr9	cg15551881**	123,688,715	**1.89 (1.22, 2.56)**	1.64 (**− **0.21, 3.49)	0.081	**1.86 (1.23, 2.49)**	**7.39E-09**	0.801	N Shelf	*TRAF1*	5'UTR
chr10	cg26712428**	105,218,771	**2.31 (1.42, 3.19)**	1.51 (**− **1.14, 4.16)	0.260	**2.22 (1.39, 3.06)**	**1.77E-07**	0.572	S Shore	*CALHM1*	TSS200
chr11	cg00574958	68,607,622	**− 6.76 (− 8.56, − 4.96)**	**− 6.57 (− 10.53, − 2.62)**	**0.001**	**− 6.73 (− 8.36, − 5.10)**	**6.42E-16**	0.932	N Shore	*CPT1A*	5'UTR
chr13	cg19750657	38,935,967	**1.93 (1.28, 2.58)**	2.28 (**− **0.39, 4.96)	0.093	**1.95 (1.32, 2.58)**	**1.12E-09**	0.799	OpenSea	*UFM1*	3'UTR
chr15	cg06192883**	52,554,171	**2.64 (1.83, 3.45)**	2.77 (0.45, 5.09)	0.020	**2.66 (1.90, 3.42)**	**8.08E-12**	0.919	OpenSea	*MYO5C*	Body
chr17	cg13274938	38,493,822	**2.63 (1.68, 3.57)**	2.98 (**− **0.74, 6.70)	0.115	**2.65 (1.73, 3.56)**	**1.46E-08**	0.854	N Shelf	*RARA*	Body
chr21	cg06500161**	43,656,587	**2.93 (2.19, 3.67)**	**5.32 (2.60, 8.04)**	**0.0002**	**3.10 (2.39, 3.81)**	**1.53E-17**	0.092	S Shore	*ABCG1*	Body
chr21	cg08309687£	35,320,596	**− 1.58 (− 2.08, − 1.08)**	**− **2.24 (**− **4.53, 0.06)	0.056	**− 1.61 (-2.10, − 1.12)**	**9.62E-11**	0.579	OpenSea		Intergenic
chr21	cg27243685**	43,642,366	**3.12 (2.08, 4.16)**	**8.91 (3.96, 13.87)**	**0.001**	**3.37 (2.35, 4.39)**	**8.95E-11**	0.023	S Shelf	*ABCG1*	Body
chr22	cg09349128	50,327,986	**− 2.46 (− 3.41, − 1.50)**	**− **2.72 (**− **5.34, -0.10)	0.042	**− 2.49 (− 3.38, − 1.59)**	**4.61E-08**	0.852	N Shore		Intergenic

*Chr* Chromosome, *CI* Confidence interval, *CpG* CpG dinucleotide, *Effect* Effect size; TSS200, 0–200 bp upstream of transcription start site; TSS1500, 200–1500 bp upstream of transcription start site; UTR, untranslated region. Numbers in bold face are statistically significant

^§^ Annotation used R v.0.6.0.*IlluminaHumanMethylation450kanno.ilmn12.hg19: Annotation for Illumina's 450 k methylation arrays*

^†^ Each effect size of the CpGs in the discovery stage was at the epigenome-wide significance level (*p* < 1E-007)

a Effect size adjusted by age and leukocyte heterogeneities (CD8^+^CD28^−^CD45RA^−^ T cell, naïve CD8 T cell, plasma blast, CD4^+^ T cell, natural killer cell, monocyte, and granulocyte)

¥ *p* value for Cochran’s Q

^*^ Promoter associated

^**^ Enhancer associated

£ Enhancer and promoter associated

**Table 3:** Genome-wide scan of DNA methylation for an association with fasting level of HOMA-IR (as a continuous variable)

Chr	CpG site§	Position	Discovery	Validation		Meta-analysis			CpG context	Gene	Gene region
			Effect† (95% CI)	Effect (95% CI)	*p*	Effect (95% CI)	*p*	*p*_Q_¥			
chr1	cg05399785	3,564,031	**3.32 (2.22, 4.43)**	1.20 (**− **3.08, 5.48)	0.578	**3.19 (2.12, 4.25)**	**4.70E-09**	0.339	N Shelf	*WDR8*	Body
chr1	cg09823288	239,552,128	**− 1.52 (− 2.02, − 1.02)**	**− 3.51 (− 5.47, -1.56)**	**0.001**	**− 1.64 (− 2.13, − 1.16)**	**1.93E− 11**	0.050	S Shore		Intergenic
chr1	cg11832534	3,563,998	**3.84 (2.47, 5.21)**	2.30 (**− **2.18, 6.78)	0.311	**3.70 (2.40, 5.01)**	**2.67E-08**	0.513	N Shelf	*WDR8*	Body
chr1	cg14476101	120,255,992	**− 1.77 (− 2.25, − 1.30)**	**− 3.73 (− 5.65, − 1.80)**	**0.0002**	**− 1.89 (− 2.35, − 1.43)**	**1.08E-15**	0.050	S Shore	*PHGDH*	Body
chr1	cg16246545	120,255,941	**− 1.91 (− 2.54, − 1.28)**	**− **3.01 (**− **5.39, **− **0.63)	0.014	**− 1.99 (− 2.60, − 1.38)**	**1.77E-10**	0.374	S Shore	*PHGDH*	Body
chr1	cg16395997	3,562,798	**2.18 (1.39, 2.96)**	2.45 (**− **0.54, 5.44)	0.107	**2.19 (1.43, 2.95)**	**1.60E-08**	0.860	S Shore	*WDR8*	Body
chr1	cg17901584*	55,353,706	**− 2.22 (− 2.94, − 1.49)**	**− **3.01 (**− **5.66, **− **0.36)	0.027	**− 2.27 (− 2.97, − 1.58)**	**1.85E-10**	0.568	S Shore	*DHCR24*	TSS1500
chr1	cg19693031	145,441,552	**− 2.66 (-3.26, − 2.07)**	**− 4.49 (− 6.37, − 2.62)**	** < 0.001**	**− 2.83 (− 3.40, − 2.27)**	**9.98E-23**	0.065	OpenSea	*TXNIP*	3'UTR
chr2	cg00391067	39,145,400	**2.93 (1.86, 4.01)**	4.25 (0.07, 8.43)	0.046	**3.02 (1.98, 4.05)**	**1.20E-08**	0.544	OpenSea	*LOC100271715*	TSS1500
chr3	cg27275941**	15,751,015	**− 2.10 (− 2.89, − 1.31)**	**− **0.55 (**− **3.60, 2.50)	0.721	**− 2.00 (− 2.77, − 1.24)**	**2.77E-07**	0.327	OpenSea	*ANKRD28*	Body
chr4	cg06690548	139,162,808	**− 1.80 (− 2.40, − 1.20)**	**− **4.25 (**− **6.83, **− **1.66)	0.002	**− 1.93 (− 2.51, − 1.34)**	**8.51E-11**	0.067	OpenSea	*SLC7A11*	Body
chr5	cg26403843	158,634,085	**1.70 (1.06, 2.34)**	1.03 (**− **0.76, 2.81)	0.257	**1.62 (1.02, 2.22)**	**1.34E-07**	0.482	N Shelf	*RNF145*	Body
chr6	cg13123009	31,681,882	**3.34 (2.16, 4.52)**	2.74 (**− **0.30, 5.79)	0.077	**3.26 (2.16, 4.36)**	**6.17E-09**	0.718	OpenSea	*LY6G6E*	TSS200
chr6	cg17501210	166,970,252	**− 1.86 (-2.49, − 1.23)**	**− 5.21 (− 8.07, -2.36)**	**0.0005**	**− 2.02 (− 2.64, − 1.41)**	**1.18E-10**	0.023	OpenSea	*RPS6KA2*	Body
chr7	cg01676795**	75,586,348	**1.92 (1.19, 2.65)**	0.96 (**− **2.66, 4.59)	0.599	**1.88 (1.16, 2.60)**	**2.59E-07**	0.608	OpenSea	*POR*	Body
chr7	cg19390658*	30,636,176	**− 1.53 (-2.07, − 1.00)**	**− **1.29 (**− **3.04, 0.46)	0.146	**− 1.51 (− 2.02, − 1.00)**	**6.86E-09**	0.792	S Shore	*GARS*	Body
chr7	cg21429551*	30,635,762	**− 1.28 (− 1.76, -0.80)**	**− **1.43 (**− **2.94, 0.09)	0.065	**− 1.29 (− 1.75, − 0.84)**	**2.29E-08**	0.855	S Shore	*GARS*	Body
chr8	cg20907614**	29,914,963	**− 1.97 (-2.72, − 1.22)**	**− **0.73 (**− **3.53, 2.06)	0.602	**− 1.88 (− 2.60, -1.16)**	**3.35E-07**	0.397	OpenSea		Intergenic
chr10	cg01391548**	97,068,696	**− 2.18 (− 2.99, − 1.37)**	**− **2.73 (**− **4.79, **− **0.67)	0.010	**− 2.25 (− 3.01, − 1.50)**	**4.43E-09**	0.623	OpenSea		Intergenic
chr10	cg02556345	124,181,965	**− 2.78 (-3.83, − 1.72)**	**− **2.20 (**− **5.91, 1.50)	0.241	**− 2.73 (− 3.74, -1.72)**	**1.22E-07**	0.767	OpenSea	*PLEKHA1*	Body
chr10	cg08284263**	92,958,627	**− 3.57 (− 4.90, − 2.24)**	**− **1.77 (**− **5.73, 2.19)	0.376	**− 3.38 (− 4.64, − 2.12)**	**1.46E-07**	0.392	OpenSea		Intergenic
chr10	cg08994060	6,214,026	**− 2.02 (− 2.78, − 1.27)**	**− **3.25 (**− **6.05, **− **0.46)	0.023	**− 2.11 (− 2.83, − 1.38)**	**1.35E-08**	0.398	OpenSea	*PFKFB3*	Body
chr10	cg26262157	6,214,079	**− 2.13 (− 2.92, − 1.34)**	**− **3.45 (**− **6.11, **− **0.79)	0.012	**− 2.24 (− 2.99, − 1.48)**	**5.89E-09**	0.344	OpenSea	*PFKFB3*	Body
chr11	cg00574958	68,607,622	**− 9.68 (− 11.83, − 7.53)**	**− 8.83 (− 13.70, − 3.96)**	**0.001**	**− 9.54 (− 11.50, − 7.58)**	**1.65E− 21**	0.751	N Shore	*CPT1A*	5'UTR
chr11	cg17058475	68,607,737	**− 4.52 (− 6.06, − 2.98)**	− 5.53 (− 9.14, − 1.91)	0.003	**− 4.67 (− 6.09, − 3.26)**	**8.84E− 11**	0.610	N Shore	*CPT1A*	5'UTR
chr13	cg19750657	38,935,967	**2.62 (1.84, 3.40)**	2.32 (-1.02, 5.67)	0.171	**2.60 (1.85, 3.36)**	**1.71E-11**	0.863	OpenSea	*UFM1*	3'UTR
chr15	Cg06192883**	52,554,171	**3.39 (2.41, 4.37)**	3.94 (1.08, 6.80)	0.008	**3.45 (2.53, 4.37)**	**2.31E-13**	0.718	OpenSea	*MYO5C*	Body
chr16	cg26663590	28,959,310	**2.27 (1.44, 3.09)**	3.59 (0.24, 6.95)	0.036	**2.35 (1.55, 3.15)**	**9.35E-09**	0.446	S Shore		Intergenic
chr17	cg13274938	38,493,822	**3.22 (2.07, 4.36)**	3.78 (-0.85, 8.41)	0.109	**3.25 (2.14, 4.36)**	**9.39E-09**	0.815	N Shelf	*RARA*	Body
chr17	cg18772573	71,257,980	**2.64 (1.63, 3.65)**	3.13 (-1.69, 7.95)	0.201	**2.66 (1.67, 3.65)**	**1.35E-07**	0.845	OpenSea	*CPSF4L*	1stExon
chr17	cg24366564**	2,843,149	**2.84 (1.74, 3.93)**	-0.91 (-4.26, 2.44)	0.589	**2.47 (1.43, 3.50)**	**3.26E-06**	0.035	OpenSea	*RAP1GAP2*	Body
chr21	cg06500161**	43,656,587	**3.75 (2.86, 4.65)**	**7.01 (3.66, 10.36)**	** < 0.001**	**3.97 (3.11, 4.83)**	**1.32E-19**	0.062	S Shore	*ABCG1*	Body
chr21	cg08309687£	35,320,596	**-2.10 (-2.70, -1.49)**	-3.34 (-6.17, -0.51)	0.021	**-2.15 (-2.74, -1.56)**	**7.59E-13**	0.392	OpenSea		Intergenic
chr21	cg27243685**	43,642,366	**3.71 (2.45, 4.97)**	**10.83 (4.64, 17.02)**	**0.001**	**4.00 (2.77, 5.24)**	**2.13E-10**	0.025	S Shelf	*ABCG1*	Body
chr22	cg09349128	50,327,986	**-3.15 (-4.30, -2.01)**	-3.71 (-6.95, -0.46)	0.026	**-3.22 (-4.30, -2.14)**	**5.31E-09**	0.749	N Shore		Intergenic

*Chr* chromosome, *CI* Confidence interval, *CpG* CpG dinucleotide, *Effect* Effect size; HOMA-IR, homeostatic model assessment–insulin resistance; TSS200, 0–200 bp upstream of transcription start site; TSS1500, 200–1500 bp upstream of transcription start site; UTR, untranslated region. Numbers in bold face are statistically significant

^§^ Annotation used R v.0.6.0.*IlluminaHumanMethylation450kanno.ilmn12.hg19: Annotation for Illumina's 450 k methylation arrays*

^†^ Each effect size of the CpGs in the discovery stage was at the epigenome-wide significance level (*p* < 1E-007)

a Effect size adjusted by age and leukocyte heterogeneities (CD8^+^CD28^−^CD45RA^−^ T cell, naïve CD8 T cell, plasma blast, CD4^+^ T cell, natural killer cell, monocyte, and granulocyte)

¥ *p* value for Cochran’s Q

^*^ Promoter associated

^**^ Enhancer associated

£ Enhancer and promoter associated

Further, we conducted a subset analysis by selecting CpGs with > 5% of a mean difference in DNAm by IR phenotypes and compared their mean differences in DNAm levels by each IR phenotype across chromosome (Chr), CpG context, enhancer and/or promoter, and gene region (Additional file 1: Figure S1). The mean levels of DNAm by FG (< 100 mg/dl vs. ≥ 100 mg/dl) differed in Chr 1, 7, 8, and 16. The mean levels of DNAm by FI (≤ 8.6μIU/ml vs. > 8.6μIU/ml) and those of DNAm by HOMA-IR (< 3.0 vs. ≥ 3.0) were different in Chr 1, 7, and 8 and in Chr 4, 8, and 11, respectively. Whereas S-Shores were hypomethylated in the groups with impaired glucose metabolism measured via FG and HOMA-IR, OpenSea, N Shelf, and S Shelf were hypermethylated in those with a greater level of FI. In this group with a higher level of FI, the enhancer was hypermethylated, whereas the promoter was hypomethylated. Gene regions, including intergenic, gene body, and 5' untranslated regions (5' UTR), were hypermethylated in the groups with, respectively, greater levels of FG, FI, and HOMA-IR, but the 200–1500 bp upstream of transcription start site (TSS1500) was hypomethylated in the group with a greater level of either FI or HOMA-IR.

### Association of the detected IR-DNAm with BC risk.

With the top 20 epigenome-wide IR-DNAm, we next tested for correlation with BC risk in the 2 independent cohorts, WHI and TCGA. In the WHI cohort, several CpGs were associated with BC development; their hazard ratios were consistent across the analyses both with and without adjustment for DM, BMI, and WHR (Table 4). In particular, 3 CpGs in *WDR8* were detected across overall, estrogen receptor/progesterone receptor–positive (ER/PR +), and human epidermal growth factor receptor-2–negative (HER2/neu–) subtypes, with a positive association with BC risk (Table 4, Additional file 1: Figure S2). None of the CpGs replicated in the analysis of IR phenotypes were detected in the analysis for BC risk, but 2 epigenome-wide level CpGs detected in discovery (cg17058475 and cg16246545) in *CPT1A* and *PHGDH* (replicated genes in relation to IR phenotypes), respectively, were associated with the risk of BC.

**Table 4:** WHI: Differentially DNA-methylated CpGs in IR significantly associated with an invasive BC risk, overall and stratified by BC molecular subtype

Chr	CpG site§	Position	Crude		DM and BMI adjusted		DM, BMI, and WHR adjusted		CpG context	Gene	Gene region
			HR (95% CI)	*p*	HR (95% CI)	*p*	HR (95% CI)	*p*			
*Overall*											
chr1	cg05399785	3,564,031	1.43 (1.05, 1.94)	0.022	1.42 (1.04, 1.93)	0.027	1.43 (1.05, 1.95)	0.023	N Shelf	*WDR8*	Body
chr1	cg11832534	3,563,998	1.35 (1.03, 1.76)	0.030	1.34 (1.02, 1.76)	0.035	1.35 (1.03, 1.78)	0.030	N Shelf	*WDR8*	Body
chr1	cg16395997	3,562,798	1.49 (1.05, 2.10)	0.026	1.49 (1.04, 2.11)	0.028	1.50 (1.05, 2.13)	0.024	S Shore	*WDR8*	Body
chr1	cg16246545	120,255,941	0.76 (0.60, 0.96)	0.021	0.77 (0.61, 0.97)	0.028	0.76 (0.60, 0.97)	0.025	S Shore	*PHGDH*	Body
chr6	cg01254034	28,543,667	0.80 (0.66, 0.98)	0.033	0.79 (0.64, 0.97)	0.023	0.79 (0.64, 0.96)	0.021	OpenSea	*SCAND3*	Body
chr16	cg05807722	67,697,020	0.81 (0.65, 1.00)	0.054	0.80 (0.65, 1.00)	0.048	0.81 (0.65, 1.00)	0.049	S Shore	*C16orf48*	3'UTR
chr17	cg24366564*	2,843,149	1.39 (1.07, 1.80)	0.015	1.37 (1.05, 1.79)	0.019	1.38 (1.06, 1.80)	0.018	OpenSea	*RAP1GAP2*	Body
*ER/PR* +											
chr1	cg05399785	3,564,031	1.62 (1.16, 2.24)	0.004	1.61 (1.15, 2.23)	0.005	1.62 (1.16, 2.26)	0.004	N Shelf	*WDR8*	Body
chr1	cg11832534	3,563,998	1.48 (1.11, 1.98)	0.008	1.47 (1.10, 1.98)	0.010	1.49 (1.10, 2.00)	0.009	N Shelf	*WDR8*	Body
chr1	cg16395997	3,562,798	1.61 (1.11, 2.34)	0.013	1.61 (1.10, 2.35)	0.014	1.62 (1.11, 2.37)	0.013	S Shore	*WDR8*	Body
chr1	cg20671910	151,262,619	1.35 (1.03, 1.78)	0.031	1.35 (1.02, 1.78)	0.033	1.37 (1.04, 1.80)	0.027	OpenSea	*ZNF687*	Body
chr7	cg01676795*	75,586,348	1.72 (1.13, 2.63)	0.012	1.72 (1.12, 2.64)	0.013	1.75 (1.14, 2.69)	0.011	OpenSea	*POR*	Body
chr10	cg00541500	45,642,600	1.33 (1.01, 1.74)	0.042	1.32 (1.00, 1.73)	0.047	1.32 (1.01, 1.74)	0.044	OpenSea	*LOC100133308*	Body
chr10	cg01391548*	97,068,696	1.31 (1.00, 1.71)	0.049	1.36 (1.04, 1.78)	0.026	1.36 (1.04, 1.79)	0.026	OpenSea		Intergenic
chr17	cg24366564*	2,843,149	1.55 (1.17, 2.06)	0.003	1.53 (1.15, 2.05)	0.004	1.54 (1.16, 2.06)	0.003	OpenSea	*RAP1GAP2*	Body
*HER2/neu -*											
chr1	cg05399785	3,564,031	1.52 (1.06, 2.18)	0.022	1.49 (1.03, 2.14)	0.033	1.49 (1.03, 2.15)	0.032	N Shelf	*WDR8*	Body
chr1	cg11832534	3,563,998	1.47 (1.07, 2.02)	0.018	1.44 (1.04, 1.99)	0.028	1.44 (1.04, 2.00)	0.027	N Shelf	*WDR8*	Body
chr1	cg16395997	3,562,798	1.60 (1.06, 2.42)	0.026	1.57 (1.03, 2.40)	0.037	1.57 (1.03, 2.41)	0.036	S Shore	*WDR8*	Body
chr1	cg16246545	120,255,941	0.75 (0.57, 1.00)	0.048	0.77 (0.58, 1.02)	0.067	0.76 (0.57, 1.02)	0.065	S Shore	*PHGDH*	Body
chr8	cg20907614*	29,914,963	0.63 (0.42, 0.95)	0.028	0.66 (0.43, 1.01)	0.053	0.66 (0.43, 1.01)	0.054	OpenSea		Intergenic
chr10	cg00541500	45,642,600	1.38 (1.02, 1.87)	0.035	1.36 (1.01, 1.85)	0.044	1.37 (1.01, 1.85)	0.042	OpenSea	*LOC100133308*	Body
chr10	cg08284263*	92,958,627	1.32 (0.96, 1.82)	0.090	1.39 (1.00, 1.92)	0.047	1.38 (1.00, 1.91)	0.050	OpenSea		Intergenic
chr11	cg17058475	68,607,737	0.61 (0.42, 0.90)	0.013	0.65 (0.44, 0.97)	0.033	0.65 (0.44, 0.96)	0.030	N Shore	*CPT1A*	5'UTR
chr17	cg24366564*	2,843,149	1.66 (1.21, 2.28)	0.002	1.64 (1.19, 2.25)	0.003	1.64 (1.19, 2.27)	0.002	OpenSea	*RAP1GAP2*	Body

*BC* Breast cancer, *BMI* Body mass index, *Chr* Chromosome, *CI* Confidence interval, *CpG* CpG dinucleotide, *DM* ever been treated for diabetes mellitus; *ER/PR* + Estrogen receptor/progesterone receptor–positive, *HER2/neu–* Human epidermal growth factor receptor-2–negative, *HR* Hazard ratio, *IR* Insulin resistance, *UTR* Untranslated region, *WHI* Women’s Health Initiative, *WHR* Waist-to-hip ratio

^§^ Annotation used R v.0.6.0.*IlluminaHumanMethylation450kanno.ilmn12.hg19: Annotation for Illumina's 450 k methylation arrays*

a HR adjusted by age and leukocyte heterogeneities (CD8^+^CD28^−^CD45RA^−^ T cell, naïve CD8 T cell, plasma blast, CD4^+^ T cell, natural killer cell, monocyte, and granulocyte)

^*^ Enhancer associated

**Fig. 3:**
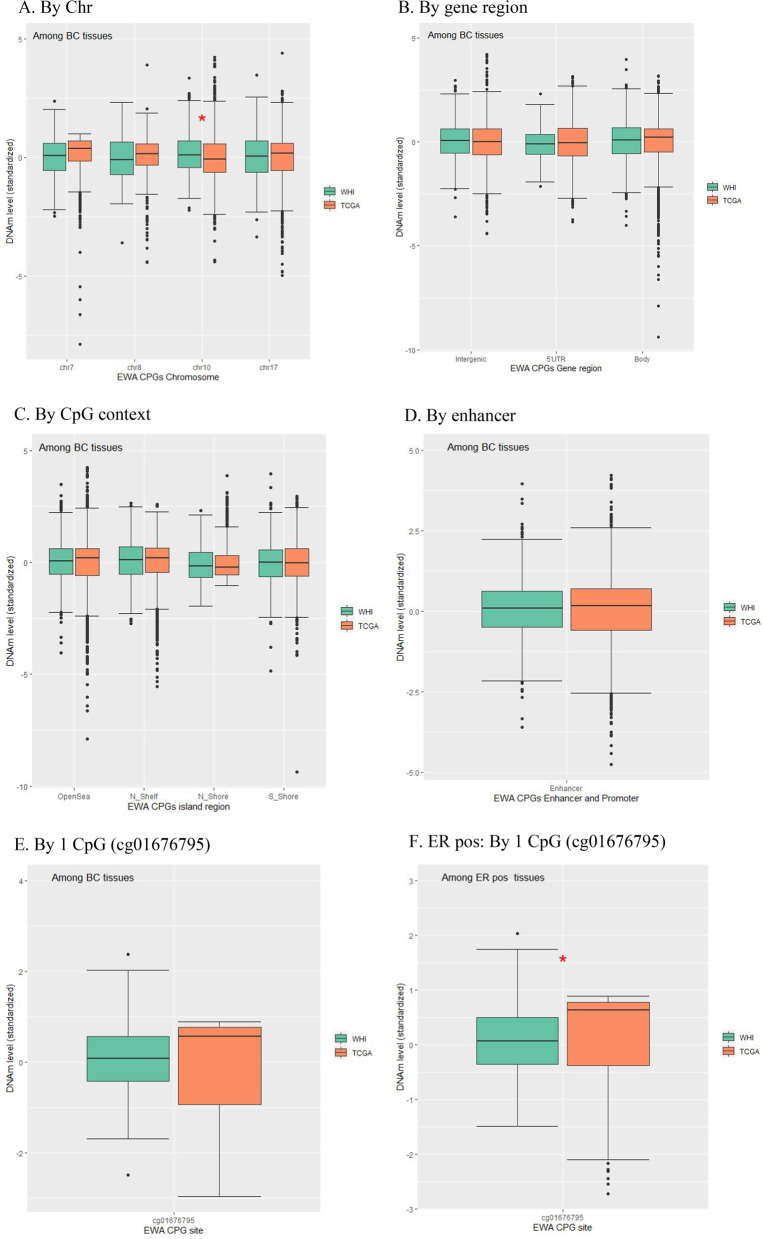
Box plots for DNAm levels from EWA IR-CpGs in association with BC, shared by BC datasets according to Chr, CpG context, and gene region. (*BC* Breast cancer, *Chr* Chromosome, *CpG* CpG dinucleotide, *DNAm* DNA methylation, *ER* pos Estrogen receptor/progesterone receptor–positive, *EWA* Epigenome-wide association, *IR* Insulin resistance, *TCGA* The Cancer Genomic Atlas, *UTR* Untranslated region, *WHI* Women’s Health Initiative*.* * Statistical significance after multiple-comparison correction). **A** By Chr; **B** By gene region; **C** By CpG context; **D** By enhancer; **E** By 1 CpG (cg01676795); **F** ER pos: By 1 CpG (cg01676795)

In the TCGA cohort, multiple CpGs were significant across BC subtypes; specifically, 2 CpGs (cg06500161 and cg27243685) in *ABCG1* (replicated gene in IR phenotypes) were significantly associated with BC risk (Table 5, Additional file 1: Figure S3). Only 1 CpG (cg01676795 in *POR*) was commonly detected across the WHI and TCGA analyses. This CpG with a 1 standardized deviation increase in DNAm had 75% (in the WHI) and a 5 times greater risk (in the TCGA) for the ER/PR + subtype. Further, we compared DNAm levels between the WHI and TCGA from the IR-CpGs associated with BC that are shared by the 2 cohorts in terms of Chr, CpGs, CpG context, and gene region. Whereas DNAm levels of some CpG contexts and/or gene regions differed significantly between the 2 cohorts among the non-BC subcohorts (Additional file 1: Figure S4), no significant difference in DNAm levels between the cohorts was observed within the BC subcohorts (Fig. 3), suggesting DNAm parallelisms between PBLs and tissues in IR and BC.

**Table 5:** TCGA: Differentially DNA-methylated CpGs in IR significantly associated with primary invasive BC tissues, overall and stratified by BC molecular subtype

Chr	CpG site§	Position	Overall		ER/PR +		HER2/neu -		CpG Context	Gene	Gene region
			HR (95% CI)	*p*	HR (95% CI)	*p*	HR (95% CI)	*p*			
chr2	cg00765233*	235,877,260	**0.10 (0.01, 0.42)**	**0.006**	**0.07 (0.003, 0.47)**	**0.030**	**0.11 (0.01, 0.39)**	**0.005**	OpenSea	*SH3BP4*	5'UTR
chr2	cg14386946**	179,315,284	**2.65 (1.36, 6.11)**	**0.010**	**5.40 (1.83, 26.55)**	**0.011**	**2.68 (1.36, 6.24)**	**0.010**	Island	*PRKRA*	Body
chr7	cg01676795*	75,586,348	**2.98 (1.06, 9.43)**	**0.046**	**5.18 (1.44, 43.15)**	**0.026**	**2.97 (1.10, 9.82)**	**0.039**	OpenSea	*POR*	Body
chr7	cg24207108	98,600,653	1.14 (0.68, 1.73)	0.567	**2.26 (1.09, 5.11)**	**0.034**	1.12 (0.64, 1.77)	0.653	OpenSea	*TRRAP*	Body
chr8	cg04684553	89,339,819	**25.73 (3.46, 305.72)**	**0.004**	NA	NA	**12.53 (2.68, 84.73)**	**0.004**	N Shore	*MMP16*	TSS200
chr10	cg26712428*	105,218,771	0.45 (0.16, 1.22)	0.119	**0.06 (0.01, 0.37)**	**0.006**	0.47 (0.16, 1.22)	0.116	S Shore	*CALHM1*	TSS200
chr12	cg20554753*	16,940,870	0.25 (0.04, 1.12)	0.085	**0.04 (0.002, 0.32)**	**0.007**	0.26 (0.05, 1.12)	0.086	OpenSea		Intergenic
chr12	cg21565550	100,967,522	**3.81 (1.48, 12.35)**	**0.012**	**3.71 (1.23, 16.33)**	**0.042**	**4.20 (1.58, 14.28)**	**0.009**	Island	*GAS2L3*	5'UTR
chr17	cg06892217**	40,075,290	**3.05 (1.44, 7.62)**	**0.008**	**5.33 (1.96, 20.16)**	**0.004**	**3.10 (1.45, 7.83)**	**0.008**	Island	*ACLY*	TSS200
chr17	cg13274938	38,493,822	**0.23 (0.05, 0.89)**	**0.046**	**0.12 (0.02, 0.61)**	**0.016**	**0.21 (0.04, 0.82)**	**0.035**	N Shelf	*RARA*	Body
chr17	cg18772573	71,257,980	0.77 (0.26, 2.01)	0.617	**0.11 (0.004, 0.74)**	**0.044**	0.77 (0.25, 2.04)	0.626	OpenSea	*CPSF4L*	1stExon
chr19	cg01969586	45,663,415	0.36 (0.09, 1.11)	0.111	**0.08 (0.01, 0.48)**	**0.015**	0.40 (0.09, 1.15)	0.122	OpenSea	*NKPD1*	TSS200
chr19	cg15197202	10,488,965	0.66 (0.32, 1.28)	0.227	**0.35 (0.11, 0.90)**	**0.047**	0.67 (0.33, 1.30)	0.244	N Shelf	*TYK2*	Body
chr19	cg21066748	46,005,805	0.61 (0.21, 1.41)	0.283	**0.10 (0.01, 0.50)**	**0.018**	0.62 (0.22, 1.39)	0.286	N Shelf		Intergenic
chr21	cg06500161*	43,656,587	**3.80 (1.15, 15.47)**	**0.043**	1.01 (0.19, 5.51)	0.993	**3.86 (1.20, 15.78)**	**0.034**	S Shore	*ABCG1*	Body
chr21	cg08309687£	35,320,596	**0.30 (0.10, 0.87)**	**0.031**	0.28 (0.05, 1.30)	0.131	**0.30 (0.10, 0.88)**	**0.033**	OpenSea		Intergenic
chr21	cg27243685*	43,642,366	**2.64 (1.39, 5.66)**	**0.005**	2.17 (0.63, 9.31)	0.258	**2.73 (1.37, 5.80)**	**0.007**	S Shelf	*ABCG1*	Body

*BC* Breast cancer, *Chr* Chromosome, *CI* Confidence interval, *CpG* CpG dinucleotide; *ER/PR* + Estrogen receptor/progesterone receptor–positive, *HER2/neu–* Human epidermal growth factor receptor-2–negative, *HR* Hazard ratio, *IR* Insulin resistance, *NA* Not available, *TCGA* The Cancer Genomic Atlas, TSS200, 0–200 bp upstream of transcription start site, *UTR* Untranslated region. Numbers in bold face are statistically significant

^§^ Annotation used R v.0.6.0.*IlluminaHumanMethylation450kanno.ilmn12.hg19: Annotation for Illumina's 450 k methylation arrays*

aHR adjusted by age, tumor purity, and cell-type proportion (cancer epithelial cell types 1 to 5 and normal epithelial, stromal, and immune cells)

^*^ Enhancer associated

^**^ Promoter associated

£ Enhancer and promoter associated

### GSEA by IR phenotypes and by BC subtypes.

Using GSEA strategies, we conducted multiple analyses of gene ontology (GO) with biologic process, cellular component, and molecular functions; pathways with *KEGG* and *Reactome*; and diseases by using *DisGeNET* and *GLAD4U* databases. In regard to IR phenotypes (Additional file 1: Tables S5.1–S4.7), GO with biologic process identified a beta-catenin/T cell factors (TCF) complex assembly; its dysregulation is associated with cancer [61]. Gene-enrichment pathways were involved in glucose intolerance, transcriptional mis-regulation in cancer, IR signaling (*AKT2*, *RSK/RAS/MAPK*), and lipid metabolism. Diseases involved in the IR pathways included nutritional and metabolic diseases, DM, and obesity. For BC subtypes, GO with a cellular component included a histone acetyltransferase and other transcription factors in the ER/PR + subtype. Genes were enriched in the pathways involving adipocytokine signaling and lipid metabolism in the HER2/neu– subtype and in those involving immune and insulin signaling (*MAPK1/MAPK3*, *Rap 1*) in both ER/PR + and HER2/neu– subtypes (Additional file 1: Tables S5.8–S4.11).

## Discussion

This is the first large population-level EWAS conducted in postmenopausal women for detecting differentially methylated CpGs in the PBLs that are associated with individual IR phenotypes and that are further prospectively evaluated for an association with BC development, both overall and in BC molecular subtypes. The methylation levels of the detected CpGs in IR and BC risk between the PBLs and the BC tissues were comparable, consistent with the findings of a gene-methylation parallelism study in glucose metabolism between peripheral blood cells and tissues [41]. This suggests that PBLs may serve as the best source of surrogate DNAm markers in non-invasive tissues, reflecting multiple interconnected glucometabolic carcinogenesis pathways.

Several EWA-CpGs in IR phenotypes detected in our study were also reported in previous studies, supporting our study’s replication and robustness. For example, cg19693031 in *TXNIP*, inversely associated with FG, FI, and IR in our study, was observed in previous studies with the same direction of association [62–66]. Thioredoxin-interacting protein (TXNIP) plays a key role in pancreatic beta cell biology involving oxidative stress and endothelial cell inflammation and its vascular complications [67], and it regulates glucose homeostasis by promoting fructose absorption in the small intestine [68]. The *TXNIP* gene is activated in both hyperglycemic animals and human adipose tissues [69], and it regulates glucometabolic pathways in human skeletal muscle [70]. Thus, our finding of hypermethylated DNA probe in *TXNIP* (i.e., a negative effect on the gene expression) associated with decreased IR has been supported.

Also, cg00574957 in *CPT1A* was negatively associated with FI and IR in both our and previous studies [63, 64, 71], showing the biological plausibility of its association with IR, including its role in obesity, metabolic syndrome, and fatty acid metabolism [72]. As this CpG is independent of nearby single-polymorphism nucleotides (SNPs) located within 1 Mb upstream or downstream of this locus, representing rs1369 index, the decreased *CPT1A* expression can be caused solely by increased methylation at this CpG site [73]. CPT1A, 1 of the 3 isoforms of CPT-1, was found mostly in the liver, where it is involved in the regulation of mitochondrial fatty acid oxidation (FAO). CPT1A deficiency causes the metabolic disorder of FAO [74, 75]. A decrease in mitochondrial fatty acid uptake results in elevated intramuscular lipid levels, but upregulates glucose oxidation and improves whole-body insulin sensitivity in a mouse model [74]; this is supportive of our finding of an inverse association between increased DNAm of the CpG (i.e., reduced gene expression) and FI/IR. However, most human gene studies have reported that this gene’s function is connected to fatty acid metabolism, not to clinical glucometabolic phenotypes, warranting a future functional study.

Similarly, cg14476101 in *PHGDH* was inversely associated with FI and IR in our study. Previous EWASs and Mendelian Randomization studies confirmed the association between hypermethylation at that locus and lower fatty-liver risk, T2DM, and adiposity [76, 77]. Also, the role of this CpG in regulating the blood concentration of steroid hormones was upregulated by obesity [78]. Together, these findings propose a plausible link between the *PHGDH* gene and lipid and adipocytokine metabolic pathways that can be altered by the methylation level of cg14476101.

In contrast, we found that cg06500161 in *ABCG1* was positively associated with FI and IR. This CpG site is a well-known DNAm probe associated with glucometabolic phenotypes [30, 62, 63, 79, 80], and the gene’s expression was inversely associated with the methylation level at this CpG [30, 62]. ABCG1 is a crucial regulator of cholesterol efflux from macrophages to high density lipoprotein (HDL); thus, suppressed gene activity by increased DNAm at this site can contribute to lowering the HDL level [81], which is a known independent risk factor for glucometabolic disorders. Also, the link between *ABCG1* and T2DM/glucose traits has been reported previously in both human and animal studies [82–84], supporting our finding of increased DNAm of this site’s being associated with IR phenotypes.

Of those validated IR-genes, 3 genes (*CPT1A, PHGDH,* and *ABCG1*) were further correlated with BC risk. In particular, the ABC transporter gene (*ABCG1*) expression associated with cholesterol efflux in the liver results in inhibition of cell proliferation and stimulation of cell apoptosis in BC cells [85]. This highlights a potential epigenetic link between lipid–glucometabolic alteration and BC tumorigenesis and progression that deserves further study. In our study, the detected CpGs in those 3 genes were EWA-based IR-DNAm probes, which are novel with respect to their association with BC risk.

Although the methylation levels of the CpGs in relation to IR and BC that are common across Chr, CpG contexts, and the gene regions were comparable between the WHI and TCGA cohorts, only 1 individual IR-CpG (cg01676795 in *POR*) was common in its relationship to BC risk in both cohorts. P450 oxidoreductase (POR) gene expression has been studied in few cancer types, presenting significant overall suppression of POR expression in muscle-invasive bladder cancer [86] and differentially expressed gene proteins enriched in neutrophil and T cell activation in hepatocellular carcinoma [87]; those findings support the important role of POR in carcinogenesis via alteration of the immune tumor microenvironment. Our finding of this CpG in POR in association with BC risk is novel, which calls for a future study on the methylation in this gene linked to BC by taking into account the effects of nearby SNPs.

Our analysis for BC risk in the TCGA included BC tissues and adjacent normal tissues. Different findings could result from the analysis between BC tissues and normal tissues (obtained from patients without BC), although we adjusted for tumor purity in the analysis. A few DNAm probes from the TCGA presented an extreme risk magnitude, warranting a further replication study with a larger independent dataset. To increase the comparability of analyses between the 2 cohorts, our study did not account for lifestyle factors in a comprehensive fashion and did not consider interactions with DNAm, which may affect the relationships between DNAm, IR, and BC. The validation data reflect a small fraction of the 2 ASs (BAA23; AS311) owing to the limited availability of IR phenotypes, resulting in less strong statistical power. In addition, given that each AS had its own study purpose, samples selected for our study may not fully represent the source population. Finally, our study population was confined to white postmenopausal women, so the generalizability of our results to other populations is limited.

## Conclusions

In conclusion, we found several differentially methylated CpGs, which are both well-established and novel, at the epigenome-wide level in relation to IR that were further correlated with BC development. Our findings warrant further validation in larger, independent epigenetic and mechanistic studies. Our study contributes to better understanding of the interconnected molecular pathways on the methylome between glucose intolerance and BC carcinogenesis and suggests the potential use of DNAm markers in PBLs as preventive targets for detecting an at-risk group for IR and BC among postmenopausal women.

## Supplementary Information

**Additional file 1:** Additional tables and figures.

## Data Availability

The data that support the findings of this study are available in accordance with policies developed by the NHLBI and WHI in order to protect sensitive participant information and approved by the Fred Hutchinson Cancer Research Center, which currently serves as the IRB of record for the WHI. Data requests may be made by emailing helpdesk@WHI.org.
