# Supplementary material for: DNA methylation in peripheral blood leukocytes for the association with glucose metabolism and invasive breast cancer
Source: Clin Epigenetics. 2023 Feb 13;15:23. doi: 10.1186/s13148-023-01435-7 (PMC9926571; doi:10.1186/s13148-023-01435-7)

Table S1. Summary of the leukocyte proportions

	CD8T	CD4T	Natural killer cell	B cell	monocyte	granulocyte
	Mean (SD)	*Mean (SD)*	*Mean (SD)*	*Mean (SD)*	*Mean (SD)*	*Mean (SD)*
Discovery dataset	9.50 (7.64)	21.30 (7.26)	2.70 (2.69)	4.30 (3.54)	12.90 (3.27)	52.80 (12.93)
Validation dataset	4.90 (4.98)	21.70 (7.92)	9.70 (5.87)	8.20 (3.41)	8.90 (3.04)	51.90 (13.27)

Table S2. Genome-wide scan of DNA methylation for an association with fasting glucose concentrations (as a categorical variable)

			Discovery	Validation		Meta-analysis					
Chr	CpG site§	Position	OR†¶ (95% CI)	OR¶ (95% CI)	P	OR (95% CI)	P	P_Q¥	CpG context	Gene	Gene region
Chr1	cg19693031	145441552	**0.37 (0.27, 0.49)**	**0.09 (0.02, 0.28)**	**9.70E-05**	**0.34 (0.26, 0.45)**	**3.77E-14**	0.027	OpenSea	*TXNIP*	3'UTR
Chr11	cg00574958	68607622	**0.36 (0.26, 0.47)**	0.71 (0.25, 1.94)	0.505	**0.37 (0.28, 0.50)**	**6.44E-12**	0.197	N Shore	*CPT1A*	5'UTR

Chr, chromosome; CI, confidence interval; CpG, CpG dinucleotide; OR, odds ratio; UTR, untranslated region. Numbers in bold face are statistically significant.

§ Annotation used R v.0.6.0.*IlluminaHumanMethylation450kanno.ilmn12.hg19: Annotation for Illumina's 450k methylation arrays.*

† Each effect size of the CpGs in the discovery stage was at the epigenome-wide significance level (p < 1E-007).

¶ Effect size adjusted by age and leukocyte heterogeneities ($CD8^{+}CD28^{-}CD45RA^{-}$ T cell, naïve CD8 T cell, plasma blast, $CD4^{+}$ T cell, natural killer cell, monocyte, and granulocyte).

¥ p value for Cochran's Q.

Table S3. Genome-wide scan of DNA methylation for an association with fasting insulin concentrations (as a categorical variable)

			Discovery	Validation		Meta-analysis					
Chr	CpG site§	Position	OR†¶ (95% CI)	OR¶ (95% CI)	P	OR (95% CI)	P	P_Q¥	CpG context	Gene	Gene region
Chr11	cg00574958	68607622	**0.50 (0.38, 0.65)**	**0.30 (0.10, 0.80)**	**0.019**	**0.48 (0.38, 0.62)**	**1.87E-08**	0.332	N Shore	*CPT1A*	5'UTR
Chr15	cg06192883*	52554171	**2.25 (1.67, 3.02)**	1.49 (0.51, 4.31)	0.463	**2.18 (1.64, 2.89)**	**7.56E-08**	0.460	OpenSea	*MYO5C*	Body

Chr, chromosome; CI, confidence interval; CpG, CpG dinucleotide; OR, odds ratio; UTR, untranslated region. Numbers in bold face are statistically significant.

§ Annotation used R v.0.6.0.*IlluminaHumanMethylation450kanno.ilmn12.hg19: Annotation for Illumina's 450k methylation arrays.*

† Each effect size of the CpGs in the discovery stage was at the epigenome-wide significance level (p < 1E-007).

¶ Effect size adjusted by age and leukocyte heterogeneities ($CD8^+CD28^-CD45RA^-$ T cell, naïve CD8 T cell, plasma blast, $CD4^+$ T cell, natural killer cell, monocyte, and granulocyte).

¥ P value for Cochran's Q.

* Enhancer associated.

Table S4. Genome-wide scan of DNA methylation for an association with fasting level of HOMA-IR (as a categorical variable)

			Discovery	Validation		Meta-analysis					
Chr	CpG site§	Position	OR†¶ (95% CI)	OR¶ (95% CI)	P	OR (95% CI)	P	P_Q¥	CpG context	Gene	Gene region
Chr1	cg14476101	120255992	**0.43 (0.32, 0.58)**	0.70 (0.22, 2.23)	0.544	**0.63 (0.49, 0.81)**	**2.86E-04**	0.840	S Shore	*PHGDH*	Body
Chr1	cg19693031	145441552	**0.42 (0.32, 0.56)**	0.30 (0.10, 0.86)	0.029	**0.62 (0.48, 0.80)**	**2.18E-04**	0.174	OpenSea	*TXNIP*	3'UTR
Chr6	cg01254034	28543667	**2.10 (1.58, 2.79)**	1.30 (0.47, 3.62)	0.611	**1.42 (1.11, 1.83)**	**5.54E-03**	0.856	OpenSea	*SCAND3*	Body
Chr11	cg00574958	68607622	**0.40 (0.30, 0.53)**	0.53 (0.18, 1.50)	0.238	**0.50 (0.39, 0.65)**	**9.89E-08**	0.915	N Shore	*CPT1A*	5'UTR
Chr13	cg02714303	76334728	**0.43 (0.31, 0.58)**	0.61 (0.19, 1.88)	0.391	**0.69 (0.53, 0.90)**	**6.04E-03**	0.825	OpenSea	*LMO7*	TSS200
Chr13	cg19750657	38935967	**2.45 (1.82, 3.33)**	0.81 (0.27, 2.44)	0.705	**1.57 (1.20, 2.05)**	**9.35E-04**	0.222	OpenSea	*UFM1*	3'UTR
Chr21	cg06500161*	43656587	**2.09 (1.58, 2.77)**	1.87 (0.65, 5.66)	0.252	**1.83 (1.42, 2.35)**	**2.27E-06**	0.966	S Shore	*ABCG1*	Body

Chr, chromosome; CI, confidence interval; CpG, CpG dinucleotide; HOMA-IR, homeostatic model assessment–insulin resistance; OR, odds ratio; TSS200, 0–200 bp upstream of transcription start site; UTR, untranslated region. Numbers in bold face are statistically significant.

§ Annotation used R v.0.6.0.*IlluminaHumanMethylation450kanno.ilmn12.hg19: Annotation for Illumina's 450k methylation arrays.*

† Each effect size of the CpGs in the discovery stage was at the epigenome-wide significance level (p < 1E-007).

¶ Effect size adjusted by age and leukocyte heterogeneities ($CD8^+CD28^-CD45RA^-$ T cell, naïve CD8 T cell, plasma blast, $CD4^+$ T cell, natural killer cell, monocyte, and granulocyte).

¥ P value for Cochran's Q.

* Enhancer associated.

Method of Interest - Over-Representation Analysis (ORA)

	Glu (n=35)<br>20 cpgs from lm and 20 from lgm	Ins (n=36)<br>20 cpgs from lm and 20 from lgm	IR (n=43)<br>35 cpgs from lm and 20 from lgm	Altogether (n=84)<br>35 glu, 36 ins, and 43 ir
**GO – biological process**	**Sig** – regulation of small molecular metabolic process<br><br>**Sig** – positive regulation of catabolic process		**Sig** - regulation of small molecular metabolic process<br><br>Other metabolic process (cellular modified amino acid, steroid)	(Table S4.5) **Sig** – beta-catenin-TCF complex assembly (dysregulation ass w/ cancer, such as CRC)<br><br>Other metabolic process (regulation of small molecular metabolic process, steroid metabolic process, and positive regulation of catabolic process)
**GO – cellular component**				
**GO – molecular function**			Carbohydrate kinase activity	Carbohydrate kinase activity
**Pathway – KEGG**	Glucose biosynthesis (glycosaminoglycan biosynthesis, Fructose/mannose metabolism, AMPK signaling pathway)<br><br>Insulin resistance<br><br>Fat metabolism (Fatty acid degradation; metabolism)	(Table S4.2) Insulin resistance,<br><br>Cancers (miRNAs in cancer, Transcriptional misregulation in cancer, AML, Thyroid cancer, SCLC, Bladder cancer)<br><br>Fat (Steroid biosynthesis; Ferroptosis)	Glucose metabolism (Fructose and mannose metabolism; AMPK signaling pathway)<br><br>Insulin resistance<br><br>Fat (Steroid biosynthesis; Ferroptosis, Fatty acid degradation)	(Table S4.6) Insulin resistance; glucose uptake (AMPK signaling)<br><br>Cancer (SCLC, leukemia, Transcriptional misregulation in cancer)<br><br>Fat (Steroid biosynthesis)
**Pathway - Reactome**	Cellular signaling (AKT2 activation; Repression of WNT target genes)	(Table S4.3) Cellular signaling (RSK activation [downstream effector of RAS/MAPK, ass with cancer], WNT signaling)<br><br>Transcription factor (AP-2 family [ass with cancer], CREB; ChREBP)<br><br>Fat (Cholesterol biosynthesis)	Cellular signaling (RSK, ERK2, ERK1)<br><br>Fat (cholesterol biosynthesis, HDL)<br><br>Immune (IL-23; IL-27)	(Table S4.7) Insulin resistance cellular signaling (AKT2, RSK activation)<br><br>Cancer (AP-2 family, Formation of the beta-catenin: TCF transactivating complex)<br><br>Fat (Cholesterol biosynthesis)
**Disease – Disgenet**	Hypoketotic hypoglycemia<br><br>Auto-immune (kidney failure, chronic; Rheumatoid Arthritis)	Phyllodes Tumor (rare breast tumor)<br><br>Kidney failure, chronic	Adrenocorticotropic hormone excess<br><br>Phyllodes Tumor<br><br>Kidney Failure, Chronic	Cancer (Bladder Neoplasm)<br><br>Auto-immune (kidney failure, chronic; Rheumatoid Arthritis)
**Disease – GLAD4U**	(Table S4.1) Diabetic Nephropathies,<br>Insulin Resistance, Diabetes Mellitus, Obesity, Nutritional and Metabolic Diseases	Cancers (lymphoma, leukemia, teratocarcinoma, CNS neoplasms, brain neoplasm)	(Table S4.4) Nutritional and Metabolic Disease<br><br>Fat (Sitosterolemia)	Cancer (Hemangiosarcoma, Leukemia

Table S5.1. Gene set analysis using GLAD4U v. 2.5: diseases with enrichment of genes that display differential DNAm at the EWA significance for the FG of PBMCs

GeneSet	Description	Gene set size	Expected value	Enrichment ratio	p value	Overlapped genes
PA443382	Anoxia	211	0.250646356	11.96905492	0.001844513	*TXNIP;PFKFB3;TRIB3*
PA444626	Insulin Resistance	248	0.294598561	10.18334915	0.002920836	*TXNIP;CPT1A;TRIB3*
PA443893	Diabetic Nephropathies	98	0.116413947	17.18007203	0.005903772	*TXNIP;XYLT1*
PA166123204	bioavailability	103	0.122353434	16.34608795	0.006502142	*TXNIP;ABCG1*
PA153619780	Sitosterolemia	6	0.007127385	140.3039216	0.007107490	*ABCG1*
PA166048726	Nutritional and Metabolic Diseases	683	0.811333939	4.930152442	0.007440437	*TXNIP;CPT1A;ABCG1;TRIB3*
PA444748	Lesch-Nyhan Syndrome	7	0.008315282	120.2605042	0.008287439	*NKPD1*
PA443886	Diabetes Mellitus	367	0.435958354	6.881391249	0.008707050	*TXNIP;ABCG1;TRIB3*
PA445131	Obesity	367	0.435958354	6.881391249	0.008707050	*CPT1A;ABCG1;TRIB3*
PA443365	Angioid Streaks	8	0.009503179	105.2279412	0.009466068	*XYLT1*

DNAm, DNA methylation; EWA, epigenome-wide association; FG, fasting glucose; PBMCs, peripheral blood mononuclear cells.

Table S5.2. KEGG pathways with enrichment of genes that display differential DNAm at the EWA significance for the FI of PBMCs

GeneSet	Description	Gene set size	Expected value	Enrichment ratio	p value	Overlapped genes
hsa05202	Transcriptional misregulation in cancer	179	0.414112	7.244413	0.007447	*MYC;TRAF1;RARA*
hsa05221	Acute myeloid leukemia	64	0.148062	13.50781	0.009306	*MYC;RARA*
hsa05222	Small cell lung cancer	91	0.210526	9.50000	0.018229	*MYC;TRAF1*
hsa04931	Insulin resistance	106	0.245228	8.15566	0.024278	*RPS6KA2;CPT1A*
hsa00100	Steroid biosynthesis	17	0.039329	25.42647	0.038653	*DHCR24*
hsa05206	MicroRNAs in cancer	155	0.358589	5.577419	0.048762	*MYC;MMP16*
hsa00020	Citrate cycle (TCA cycle)	28	0.064777	15.43750	0.062913	*ACLY*
hsa04216	Ferroptosis	37	0.085599	11.68243	0.082334	*SLC7A11*
hsa05216	Thyroid cancer	37	0.085599	11.68243	0.082334	*MYC*
hsa04714	Thermogenesis	218	0.504338	3.965596	0.088852	*RPS6KA2;CPT1A*

DNAm, DNA methylation; EWA, epigenome-wide association; FI, fasting insulin; PBMCs, peripheral blood mononuclear cells.

Table S5.3. Reactome pathways with enrichment of genes that display differential DNAm at the EWA significance for the FI of PBMCs

**GeneSet**	**Description**	**Gene set size**	**Expected value**	**Enrichment ratio**	**p value**	**Overlapped genes**
R-HSA-5362517	Signaling by Retinoic Acid	42	0.070960048	28.18487395	0.002223243	*CPT1A;RARA*
R-HSA-9006931	Signaling by Nuclear Receptors	189	0.319320215	9.394957983	0.003652756	*MYC;CPT1A;RARA*
R-HSA-6807047	Cholesterol biosynthesis via desmosterol	3	0.005068575	197.2941176	0.005060518	*DHCR24*
R-HSA-6807062	Cholesterol biosynthesis via lathosterol	3	0.005068575	197.2941176	0.005060518	*DHCR24*
R-HSA-8866911	TFAP2 (AP-2) family regulates transcription of cell cycle factors	5	0.008447625	118.3764706	0.008420796	*MYC*
R-HSA-444257	RSK activation	6	0.01013715	98.64705882	0.010096927	*RPS6KA2*
R-HSA-199920	CREB phosphorylation	7	0.011826675	84.55462185	0.011770391	*RPS6KA2*
R-HSA-163765	ChREBP activates metabolic gene expression	8	0.013516200	73.98529412	0.013441192	*ACLY*
R-HSA-4411364	Binding of TCF/LEF:CTNNB1 to target gene promoters	8	0.013516200	73.98529412	0.013441192	*MYC*
R-HSA-8951430	RUNX3 regulates WNT signaling	8	0.013516200	73.98529412	0.013441192	*MYC*

DNAm, DNA methylation; EWA, epigenome-wide association; FI, fasting insulin; PBMCs, peripheral blood mononuclear cells.

Table S5.4. Gene set analysis using GLAD4U v. 2.5: diseases with enrichment of genes that display differential DNAm at the EWA significance for the IR of PBMCs

GeneSet	Description	Gene set size	Expected value	Enrichment ratio	p value	Overlapped genes
PA443882	Dermatomyositis	34	0.031972284	62.55417957	4.62E-04	*GARS;TYK2*
PA444944	Microcephaly	178	0.167384311	17.92282673	5.87E-04	*PHGDH;DHCR24;UFM1*
PA446477	Polymyositis	49	0.046077704	43.40494092	9.60E-04	*GARS;TYK2*
PA443737	Cleft Palate	232	0.218163821	13.75113430	0.001264014	*DHCR24;SLC7A11;RARA*
PA447188	Genetic Diseases, Inborn	259	0.243553576	12.31761837	0.001733051	*PHGDH;DHCR24;CPT1A*
PA166123204	bioavailability	115	0.108141549	18.49427918	0.005154861	*TXNIP;ABCG1*
PA443963	Dysostoses	117	0.110022272	18.17813765	0.005330557	*POR;SALL1*
PA446836	Craniofacial Abnormalities	388	0.364860183	8.222327727	0.005417743	*PHGDH;POR;RARA*
PA153619780	Sitosterolemia	6	0.005642168	177.2368421	0.005629615	*ABCG1*
PA166048726	Nutritional and Metabolic Diseases	822	0.772976986	5.174798310	0.006465472	*TXNIP;POR;CPT1A;ABCG1*

DNAm, DNA methylation; EWA, epigenome-wide association; IR, insulin resistance; PBMCs, peripheral blood mononuclear cells.

Table S5.5. Gerontology analysis: biological process with enrichment of genes that display differential DNAm at the EWA significance for either FG, FI, or IR of PBMCs

GeneSet	Description	Gene set size	Expected value	Enrichment ratio	p value	Overlapped genes
GO:1904837	beta-catenin-TCF complex assembly	28	0.071312345	42.06845238	4.76E-05	*TRRAP;TLE3;MYC*
GO:0062012	regulation of small molecule metabolic process	331	0.843013796	7.117321249	1.70E-04	*SLC7A11;PFKFB3;CPT1A;ABCG1;TRIB3;POR*
GO:0008202	steroid metabolic process	288	0.733498408	6.816647377	7.62E-04	*PLEKHA1;ABCG1;DHCR24;ACLY;POR*
GO:1902532	negative regulation of intracellular signal transduction	478	1.217403608	4.928521618	0.001190877	*SH3BP4;PLEKHA1;TRIB3;MYC;EPHB2;PDE3A*
GO:0060021	roof of mouth development	89	0.226671383	13.23501873	0.001482517	*PLEKHA1;EPHB2;COL11A2*
GO:0060348	bone development	200	0.509373895	7.852777778	0.001607371	*XYLT1;RARA;MMP16;POR*
GO:0048705	skeletal system morphogenesis	223	0.567951892	7.042850025	0.002390799	*PLEKHA1;RARA;MMP16;POR*
GO:0009896	positive regulation of catabolic process	395	1.006013442	4.970112518	0.003068314	*SH3BP4;PFKFB3;CPT1A;TRIM8;TRIB3*
GO:0044091	membrane biogenesis	34	0.086593562	23.09640523	0.003361377	*S100A10;EPHB2*
GO:0015711	organic anion transport	439	1.118075699	4.471969122	0.004812288	*SH3BP4;SLC7A11;CPT1A;ABCG1;CALHM1*

DNAm, DNA methylation; EWA, epigenome-wide association; FG, fasting glucose; FI, fasting insulin; IR, insulin resistance; PBMCs, peripheral blood mononuclear cells.

Table S5.6. KEGG pathways with enrichment of genes that display differential DNAm at the EWA significance for either FG, FI, or IR of PBMCs

GeneSet	Description	Gene set size	Expected value	Enrichment ratio	p value	Overlapped genes
hsa04931	Insulin resistance	106	0.367842684	8.155660377	0.005604631	*CPT1A;TRIB3;RPS6KA2*
hsa02010	ABC transporters	43	0.149219202	13.40310078	0.009556280	*ABCG1;TAP2*
hsa05221	Acute myeloid leukemia	64	0.222093696	9.005208333	0.020412008	*MYC;RARA*
hsa05168	Herpes simplex infection	172	0.596876807	5.026162791	0.020846082	*TAP2;TRAF1;TYK2*
hsa05202	Transcriptional misregulation in cancer	179	0.621168305	4.829608939	0.023143100	*MYC;TRAF1;RARA*
hsa05169	Epstein-Barr virus infection	191	0.662810873	4.526178010	0.027393285	*TAP2;MYC;TYK2*
hsa05222	Small cell lung cancer	91	0.315789474	6.333333333	0.039179463	*MYC;TRAF1*
hsa04659	Th17 cell differentiation	105	0.36437247	5.488888889	0.050733318	*RARA;TYK2*
hsa00100	Steroid biosynthesis	17	0.058993638	16.95098039	0.057448598	*DHCR24*
hsa04152	AMPK signaling pathway	116	0.402544824	4.968390805	0.060571410	*PFKFB3;CPT1A*

DNAm, DNA methylation; EWA, epigenome-wide association; FG, fasting glucose; FI, fasting insulin; IR, insulin resistance; PBMCs, peripheral blood mononuclear cells.

Table S5.7. Reactome pathways with enrichment of genes that display differential DNAm at the EWA significance for either FG, FI, or IR of PBMCs

GeneSet	Description	Gene set size	Expected value	Enrichment ratio	p value	Overlapped genes
R-HSA-201722	Formation of the beta-catenin:TCF transactivating complex	86	0.264957265	11.32258065	0.002279635	*TRRAP;TLE3;MYC*
R-HSA-9006931	Signaling by Nuclear Receptors	189	0.582289803	6.869431644	0.002551349	*CPT1A;TLE3;MYC;RARA*
R-HSA-5362517	Signaling by Retinoic Acid	42	0.129397734	15.45622120	0.007325768	*CPT1A;RARA*
R-HSA-2122947	NOTCH1 Intracellular Domain Regulates Transcription	44	0.135559531	14.75366569	0.008018290	*TLE3;MYC*
R-HSA-6807047	Cholesterol biosynthesis via desmosterol	3	0.009242695	108.1935484	0.009215162	*DHCR24*
R-HSA-6807062	Cholesterol biosynthesis via lathosterol	3	0.009242695	108.1935484	0.009215162	*DHCR24*
R-HSA-165158	Activation of AKT2	4	0.012323594	81.14516129	0.012268580	*TRIB3*
R-HSA-8866911	TFAP2 (AP-2) family regulates transcription of cell cycle factors	5	0.015404492	64.91612903	0.015312890	*MYC*
R-HSA-444257	RSK activation	6	0.018485391	54.09677419	0.018348119	*RPS6KA2*
R-HSA-1980143	Signaling by NOTCH1	69	0.212581992	9.408134642	0.018959443	*TLE3;MYC*

DNAm, DNA methylation; EWA, epigenome-wide association; FG, fasting glucose; FI, fasting insulin; IR, insulin resistance; PBMCs, peripheral blood mononuclear cells.

Method of Interest - Over-Representation Analysis (ORA)

	ER pos	HER2 neg	Altogether (ER, HER2, overall)
GO – biological process	**Sig** – skeletal system morphogenesis	**Sig** – Fat biosynthetic/metabolic process (cholesterol/sterol/steroid; carnitine metabolic process)	**Sig** – Fat biosynthetic/metabolic process (cholesterol/sterol/steroid)<br><br>Another fat process (carnitine metabolic process)
GO – cellular component	Histone acetyltransferase (STAGA/SAGA/TFTC plus NuA4/H4/H2A)<br><br>Transcription factor (mRNA/RNA polymerase II/nuclear transcription factor)		Histone acetyltransferase (STAGA/SAGA/TFTC/NuA4/PCAF [ass w/cancer, transcriptional coactivator of p53-genes])<br><br>Transcription factor (mRNA/RNA polymerase II/nuclear transcription factor)
GO – molecular function	Type 1 angiotensin receptor binding<br><br>Growth hormone receptor binding	Fat metabolic process (carnitine O-acyltransferase/O-palmitoyltransferase activity/lipid-transporting APTase activity)	Fat metabolic process (carnitine O-acyltransferase/O-palmitoyltransferase activity/lipid-transporting APTase activity)<br><br>Angiotensin receptor binding<br><br>Growth hormone receptor binding
Pathway – KEGG	Immune-related (Th17/Th1 and Th2 cell differentiation)	(Table S4.9) Adipocytokine signaling pathway; glucagon signaling<br><br>Fat (Fatty acid degradation/metabolism)<br><br>Immune (Th17cell differentiation)	Adipocytokine signaling pathway<br><br>Fat (Fatty acid degradation/metabolism)<br><br>Immune (Th17/Th1 and Th2 cell differentiation)
Pathway - Reactome	(Table S4.8) Insulin cellular signaling (MAPK1/MAPK3 activation)<br><br>Rap 1 signaling (cell invasion; metastasis in cancers)<br><br>Immune-related (IL6/IL-23/IL-27/IL-35/IFN)<br><br>Transcription factor (ChREBP activates metabolic gene expression, siRNA biogenesis)	Cellular signaling (Rap 1 signaling [playing a role in cancer])<br><br>Fat (HDL remodeling, lipid homeostasis, fatty acid metabolism)<br><br>Transcription factor (ChREBP activates metabolic gene expression, siRNA biogenesis)	(Table S4.11) Insulin cellular signaling (MAPK1/MAPK3 activation)<br><br>Immune-related (IL6/IL-23/IL-27/IL-35)<br><br>Fat (HDL remodeling, fatty acid metabolism)<br><br>Transcription factor (ChREBP activates metabolic gene expression, siRNA biogenesis)
Disease – Disgenet	Adrenocorticotropic hormone excess<br><br>Phyllodes Tumor<br><br>Neoplasms; bladder neoplasm	Adrenocorticotropic hormone excess<br><br>Phyllodes Tumor<br><br>Neoplasms; kidney failure, chronic	Adrenocorticotropic hormone excess<br><br>Cancer (Phyllodes Tumor, Neoplasms, Bladder neoplasm, Neoplastic cell transformation)
Disease – GLAD4U		(Table S4.10) Nutritional and metabolic disease	Nutritional and metabolic disease

Table S5.8. Reactome pathways with enrichment of genes that display differential DNAm shared by PBMCs (in WHI) and BC tissues (in TCGA) among ER/PR pos type

GeneSet	Description	Gene set size	Expected value	Enrichment ratio	p value	Overlapped genes
R-HSA-163765	ChREBP activates metabolic gene expression	8	0.0064	157.2188	0.0063	*ACLY*
R-HSA-9020933	Interleukin-23 signaling	9	0.0072	139.7500	0.0071	*TYK2*
R-HSA-112411	*MAPK1 (ERK2)* activation	9	0.0072	139.7500	0.0071	*TYK2*
R-HSA-110056	*MAPK3 (ERK1)* activation	10	0.0080	125.7750	0.0079	*TYK2*
R-HSA-9020956	Interleukin-27 signaling	11	0.0087	114.3409	0.0087	*TYK2*
R-HSA-1059683	Interleukin-6 signaling	11	0.0087	114.3409	0.0087	*TYK2*
R-HSA-8984722	Interleukin-35 signaling	12	0.0095	104.8125	0.0095	*TYK2*
R-HSA-392517	Rap1 signaling	15	0.0119	83.8500	0.0119	*RAP1GAP2*
R-HSA-6788467	IL-6-type cytokine receptor ligand interactions	17	0.0135	73.9853	0.0134	*TYK2*
R-HSA-912694	Regulation of IFNA signaling	22	0.0175	57.1705	0.0174	*TYK2*

BC, breast cancer; DNAm, DNA methylation; ER/PR pos, estrogen/progesterone receptor positive; IFNA, interferon alpha; PBMCs, peripheral blood mononuclear cells; TCGA, The Cancer Genome Atlas; WHI, Women’s Health Initiative.

Table S5.9. KEGG pathways with enrichment of genes that display differential DNAm shared by PBMCs (in WHI) and BC tissues (in TCGA) among HER2/neu neg type

GeneSet	Description	Gene set size	Expected value	Enrichment ratio	p value	Overlapped genes
hsa00020	Citrate cycle (TCA cycle)	28	0.0202	49.4000	0.0201	*ACLY*
hsa00071	Fatty acid degradation	41	0.0296	33.7366	0.0293	*CPT1A*
hsa02010	ABC transporters	43	0.0311	32.1674	0.0307	*ABCG1*
hsa01212	Fatty acid metabolism	45	0.0325	30.7378	0.0321	*CPT1A*
hsa05221	Acute myeloid leukemia	64	0.0463	21.6125	0.0454	*RARA*
hsa04920	Adipocytokine signaling pathway	68	0.0492	20.3412	0.0482	*CPT1A*
hsa03320	PPAR signaling pathway	72	0.0521	19.2111	0.0510	*CPT1A*
hsa04928	Parathyroid hormone synthesis, secretion and action	101	0.0730	13.6950	0.0709	*MMP16*
hsa04922	Glucagon signaling pathway	102	0.0737	13.5608	0.0716	*CPT1A*
hsa04659	Th17 cell differentiation	105	0.0759	13.1733	0.0737	*RARA*

BC, breast cancer; DNAm, DNA methylation; HER2/neu neg, human epidermal growth factor receptor 2, negative; PBMCs, peripheral blood mononuclear cells; TCGA, The Cancer Genome Atlas; WHI, Women's Health Initiative.

Table S5.10. Gene set analysis using GLAD4U v. 2.5: diseases with enrichment of genes that display differential DNAm shared by PBMCs (in WHI) and BC tissues (in TCGA) among HER2/neu neg types

GeneSet	Description	Gene set size	Expected value	Enrichment ratio	p value	Overlapped genes
PA153619780	Sitosterolemia	6	0.0034	298.1458	0.0033	*ABCG1*
PA166048726	Nutritional and Metabolic Diseases	683	0.3818	7.8574	0.0051	*ABCG1;CPT1A;POR*
PA445547	Reye Syndrome	11	0.0061	162.6250	0.0061	*CPT1A*
PA445812	Tangier Disease	14	0.0078	127.7768	0.0078	*ABCG1*
PA443286	Adrenal Hyperplasia, Congenital	22	0.0123	81.3125	0.0122	*POR*
PA446836	Craniofacial Abnormalities	319	0.1783	11.2155	0.0127	*RARA;POR*
PA165108302	Pulmonary alveolar proteinosis	23	0.0129	77.7772	0.0128	*ABCG1*
PA166128121	cardiotoxicity	23	0.0129	77.7772	0.0128	*POR*
PA165108566	Adrenogenital syndrome NOS	26	0.0145	68.8029	0.0144	*POR*
PA444432	Hepatic Encephalopathy	27	0.0151	66.2546	0.0150	*CPT1A*

BC, breast cancer; DNAm, DNA methylation; HER2/neu neg, human epidermal growth factor receptor 2, negative; PBMCs, peripheral blood mononuclear cells; TCGA, The Cancer Genome Atlas; WHI, Women's Health Initiative.

Table S5.11. Reactome pathways with enrichment of genes that display differential DNAm shared by PBMCs (in WHI) and BC tissues (in TCGA) across BC types

GeneSet	Description	Gene set size	Expected value	Enrichment ratio	p value	Overlapped genes
R-HSA-5362517	Signaling by Retinoic Acid	42	0.0376	53.2381	0.0006	*RARA;CPT1A*
R-HSA-163765	ChREBP activates metabolic gene expression	8	0.0072	139.7500	0.0071	*ACLY*
R-HSA-9020933	Interleukin-23 signaling	9	0.0081	124.2222	0.0080	*TYK2*
R-HSA-112411	MAPK1 (ERK2) activation	9	0.0081	124.2222	0.0080	*TYK2*
R-HSA-8964058	HDL remodeling	10	0.0089	111.8000	0.0089	*ABCG1*
R-HSA-110056	MAPK3 (ERK1) activation	10	0.0089	111.8000	0.0089	*TYK2*
R-HSA-8978868	Fatty acid metabolism	167	0.1494	13.3892	0.0091	*ACLY;CPT1A*
R-HSA-9020956	Interleukin-27 signaling	11	0.0098	101.6364	0.0098	*TYK2*
R-HSA-1059683	Interleukin-6 signaling	11	0.0098	101.6364	0.0098	*TYK2*
R-HSA-8984722	Interleukin-35 Signalling	12	0.0107	93.1667	0.0107	*TYK2*

BC, breast cancer; DNAm, DNA methylation; PBMCs, peripheral blood mononuclear cells; TCGA, The Cancer Genome Atlas; WHI, Women's Health Initiative.

Figure S1. Mean difference in DNAm levels of selected CpGs across chromosome, CpG context, enhancer and/or promoter, and gene region, stratified by IR status. (CpG, CpG dinucleotide; DNAm, DNA methylation; FG and FI, fasting levels of glucose and insulin; HOMA-IR, homeostatic model assessment-insulin resistance; TSS200, 0–200 bp upstream of transcription start site; TSS1500, 200–1500 bp upstream of transcription start site; UTR, untranslated region. Note: CpGs were selected with a mean difference of > 5% in DNAm by IR. * Statistical significance after multiple comparison correction.)

A. FG: By chromosome

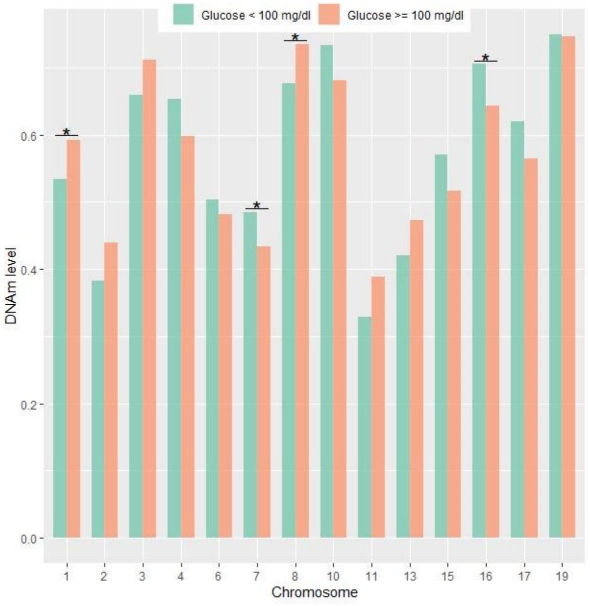

B. FG: By CpG context

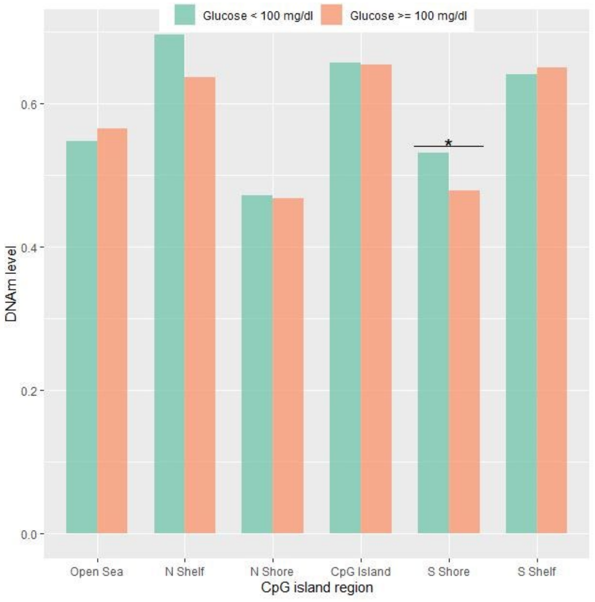

C. FG: By enhancer and promoter

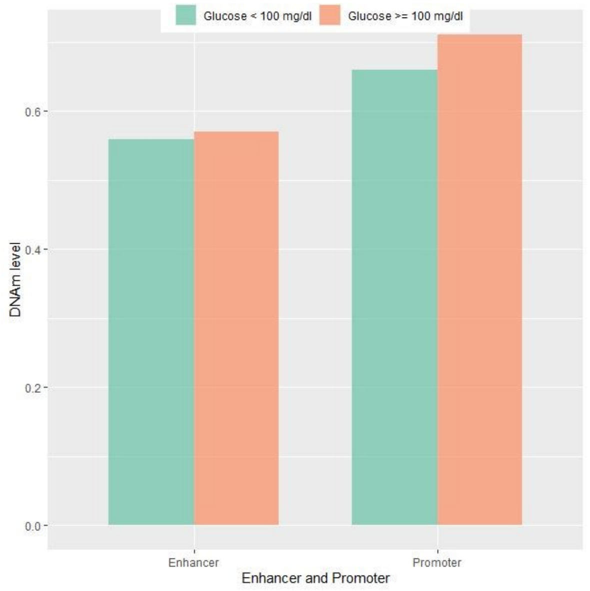

D. FG: By gene region

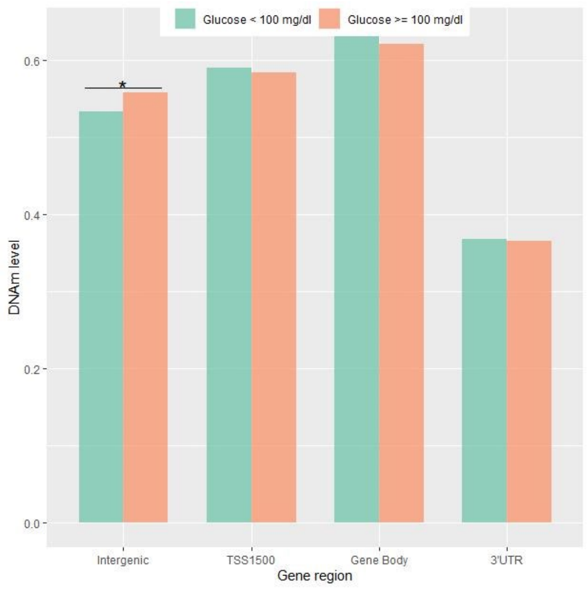

E. FI: By chromosome

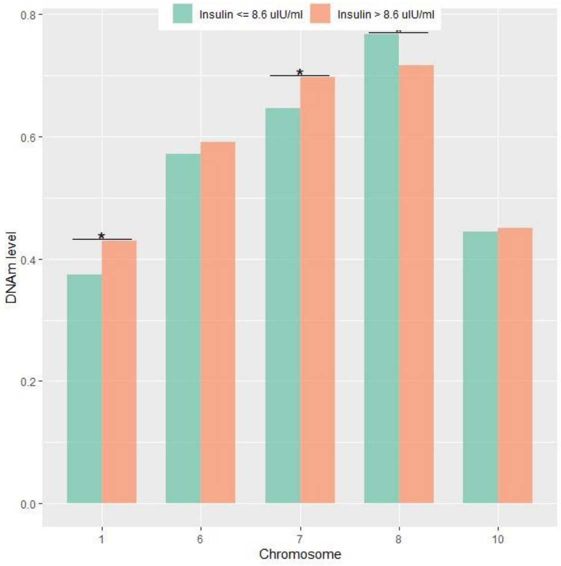

F. FI: By CpG context

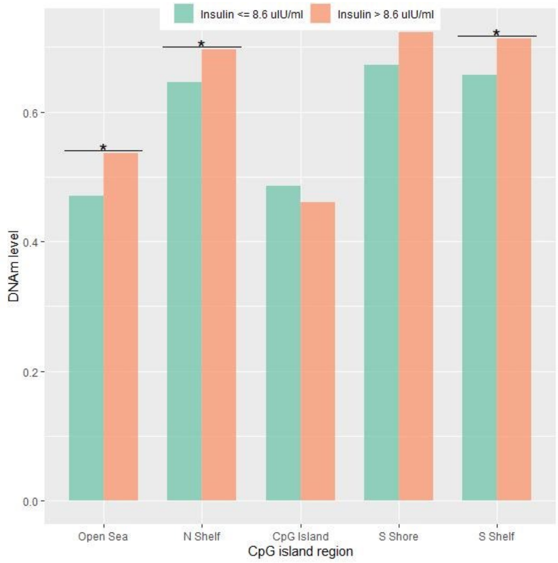

G. FI: By enhancer and promoter

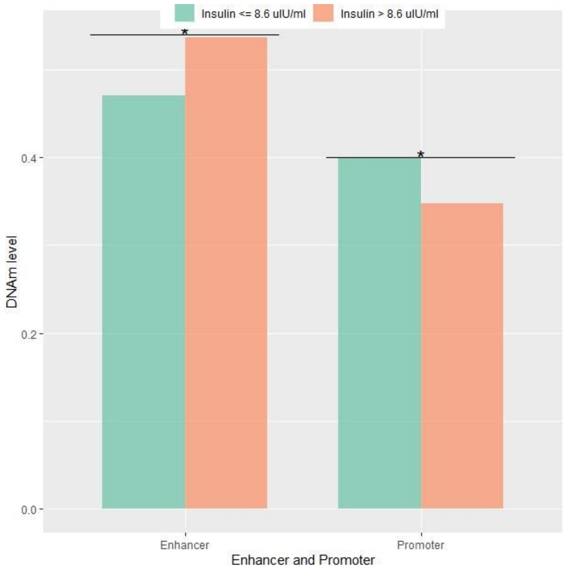

H. FI: By gene region

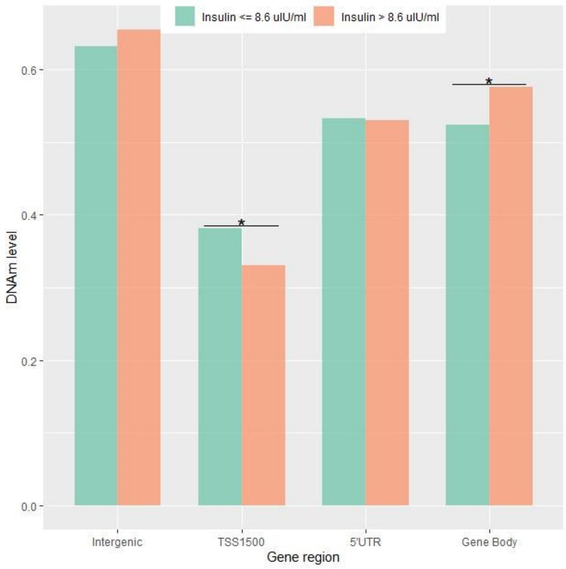

I. HOMA-IR: By chromosome

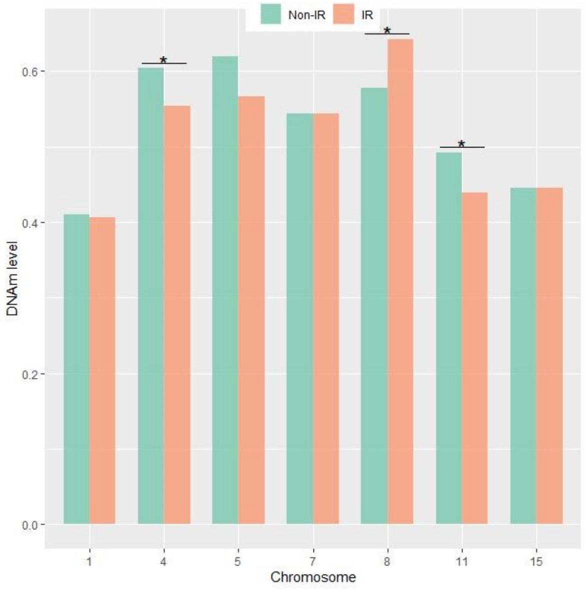

J. HOMA-IR: By CpG context

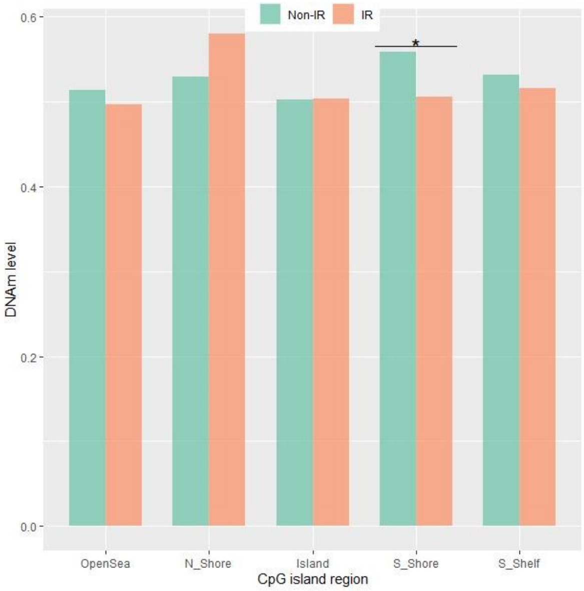

K. HOMA-IR: By enhancer and promoter

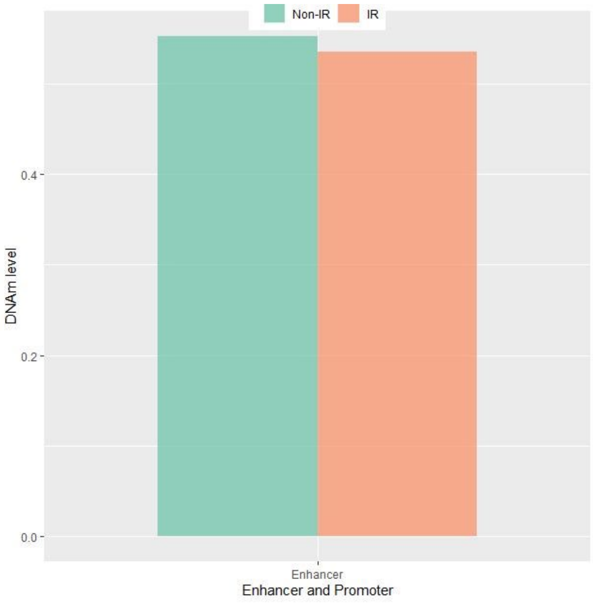

L. HOMA-IR: By gene region

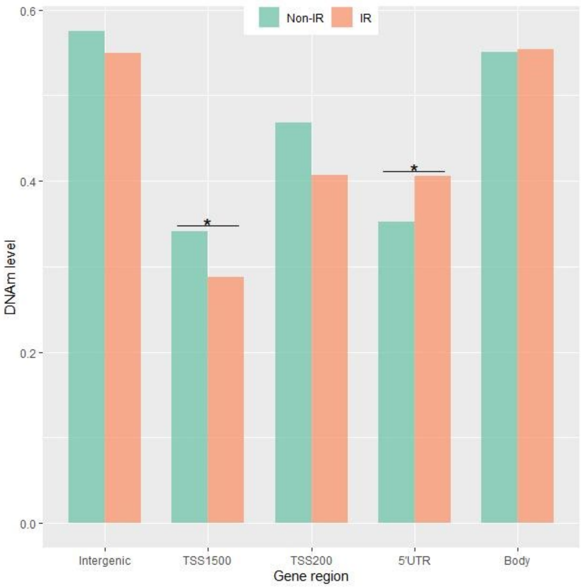

Figure S2. Box plots for DNAm levels of selected genes that are significantly different by BC status in the WHI data. (BC, breast cancer; CpG, CpG dinucleotide; DNAm, DNA methylation; ER/PR pos, estrogen receptor/progesterone receptor–positive; EWA, epigenome-wide association; HER2/neu neg, human epidermal growth factor receptor-2–negative; IR, insulin resistance; WHI, Women's Health Initiative. Note: DNAm of each gene comprises that of relevant EWA IR-CpGs in association with BC risk. * Statistical significance.)

A. Among HER2/neu neg: *CPT1A*

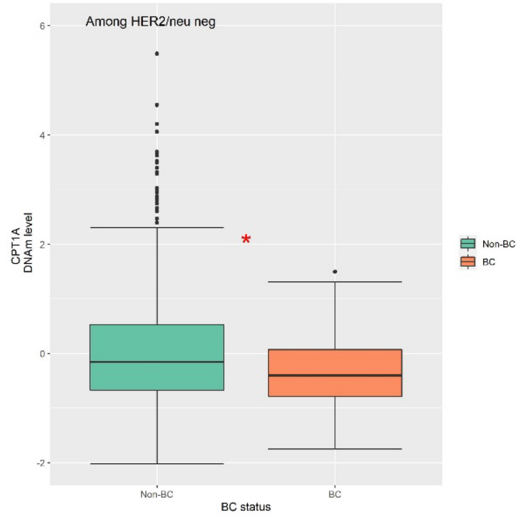

B. Overall: *WDR8*

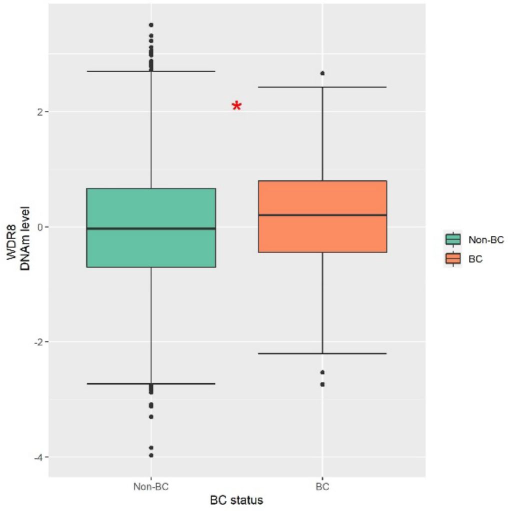

C. Among ER/PR pos: *WDR8*

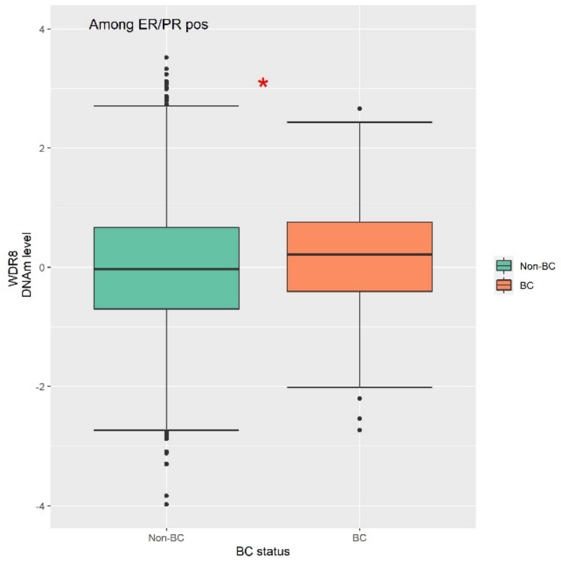

D. Among HER2/neu neg: *WDR8*

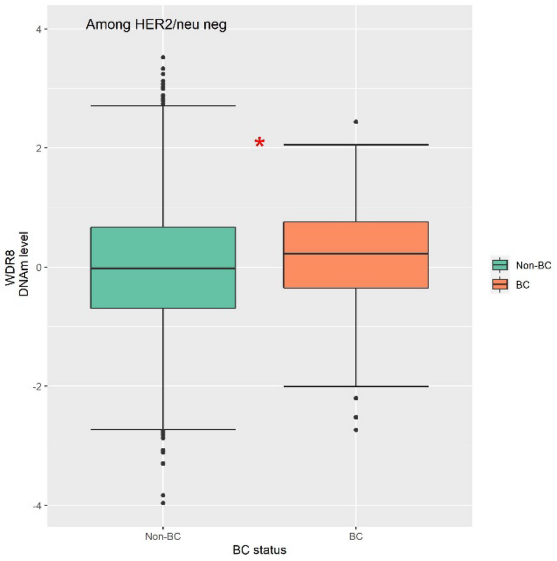

Figure S3. Box plots for DNAm levels of selected genes that are significantly different by BC status in the TCGA data. (BC, breast cancer; CpG, CpG dinucleotide; DNAm, DNA methylation; ER/PR pos, estrogen receptor/progesterone receptor–positive; EWA, epigenome-wide association; HER2/neu neg, human epidermal growth factor receptor-2–negative; IR, insulin resistance; TCGA, the Cancer Genomic Atlas. Note: DNAm of each gene comprises that of relevant EWA IR-CpGs in association with BC risk. * Statistical significance.)

A. Overall: *ACLY*

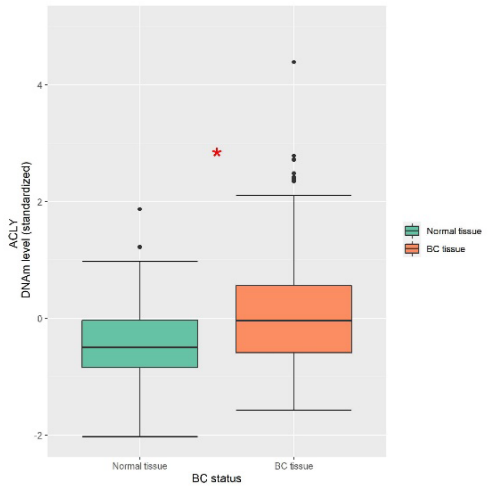

B. Among ER/PR pos: *ACLY*

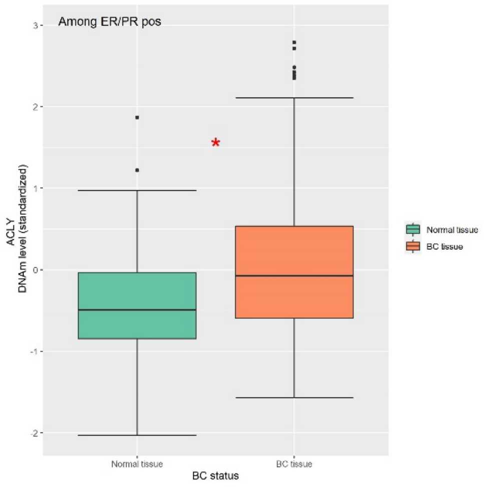

C. Among HER2/neu neg: *ACLY*

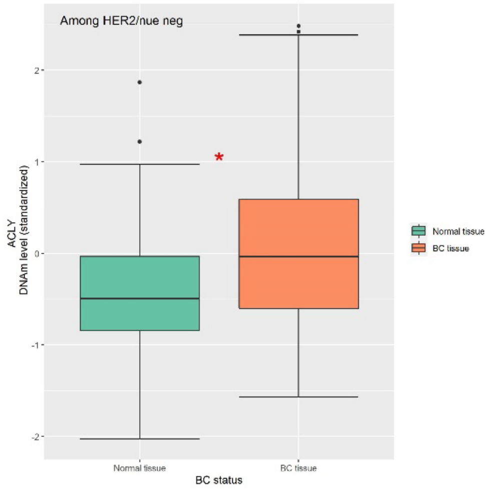

D. Overall: *ABCG1*

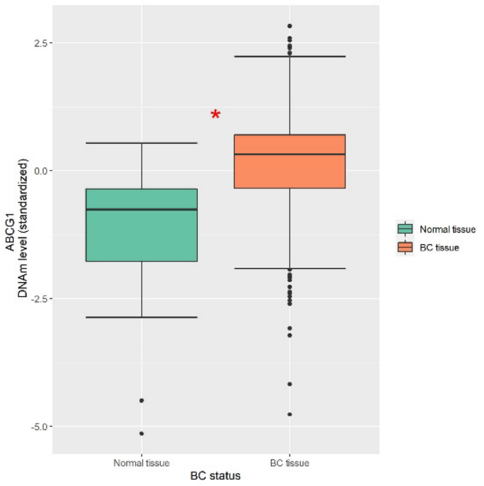

E. Among HER2/neu pos: *ABCG1*

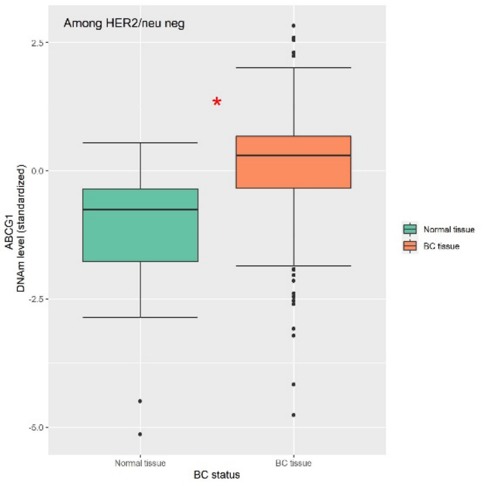

F. Among ER/PR pos: *CPSF4L*

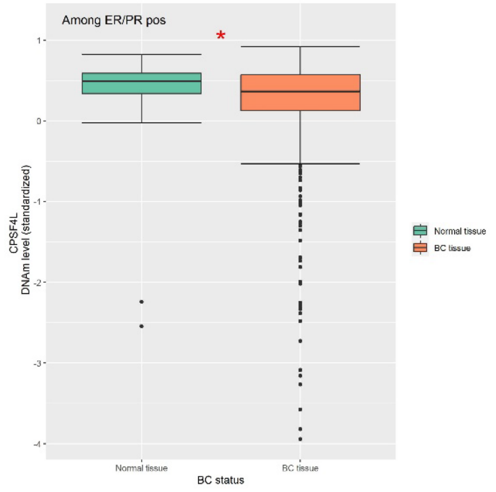

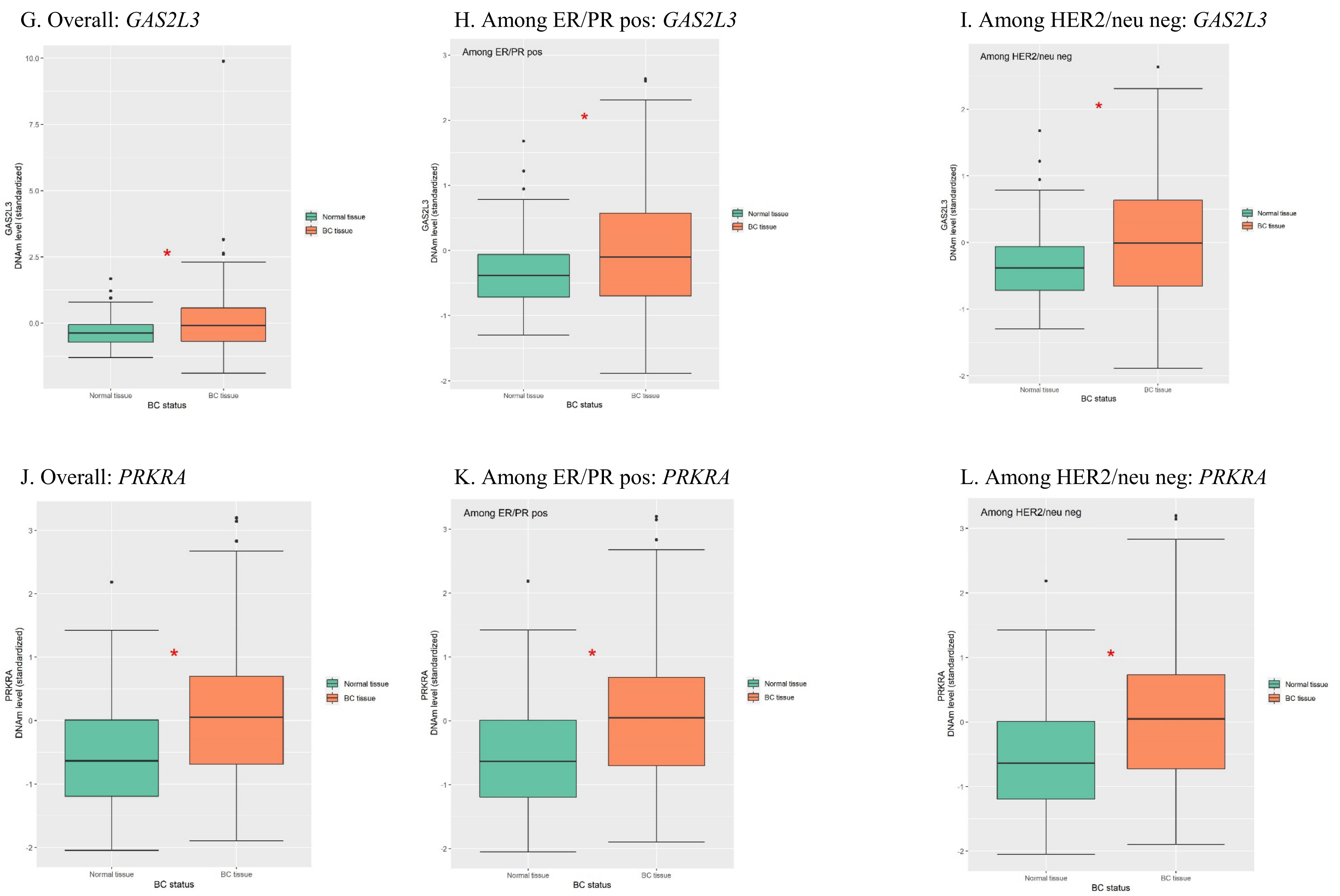

G. Overall: *GAS2L3*
H. Among ER/PR pos: *GAS2L3*
I. Among HER2/neu neg: *GAS2L3*
Among ER/PR pos
Among HER2/neu neg
GAS2L3
DNAm level (standardized)
Normal tissue
BC tissue
BC status
J. Overall: *PRKRA*
K. Among ER/PR pos: *PRKRA*
L. Among HER2/neu neg: *PRKRA*
PRKRA

M. Overall: *SH3BP4*

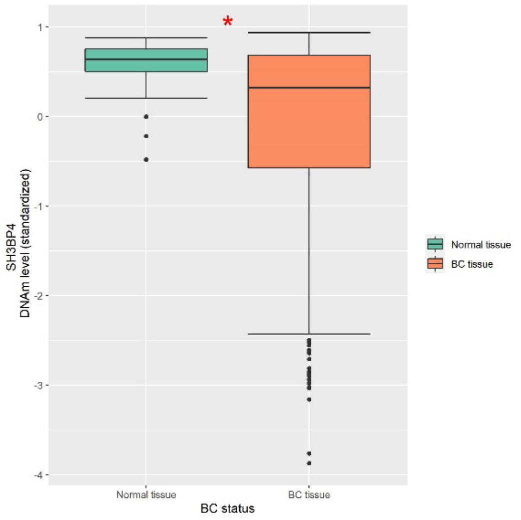

N. Among ER/PR pos: *SH3BP4*

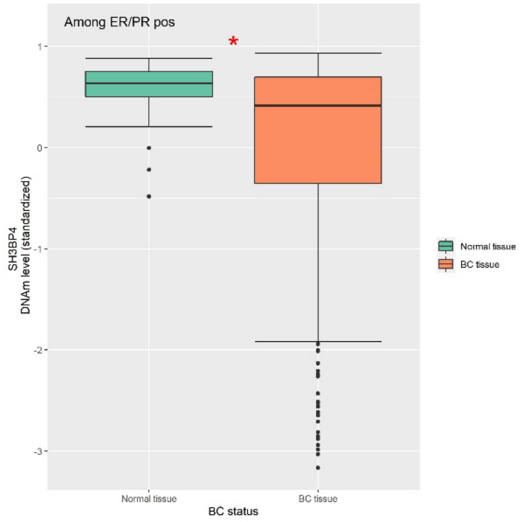

O. Among HER2/neu neg: *SH3BP4*

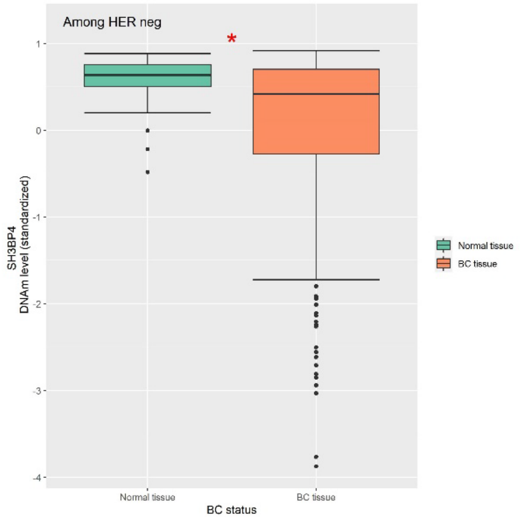

P. Among ER/PR pos: *NKPD1*

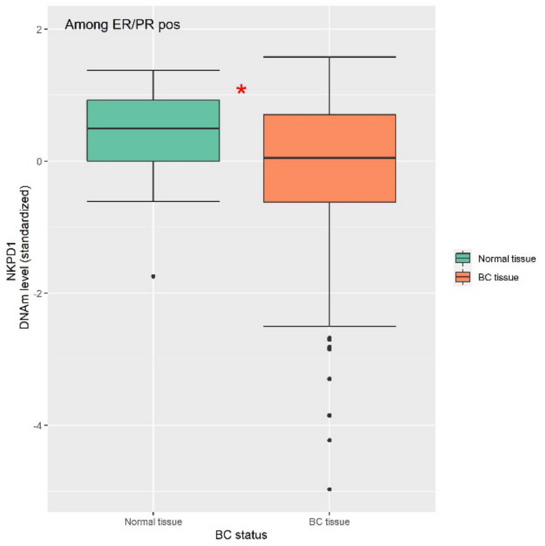

Q. Among ER/PR pos: *TRRAP*

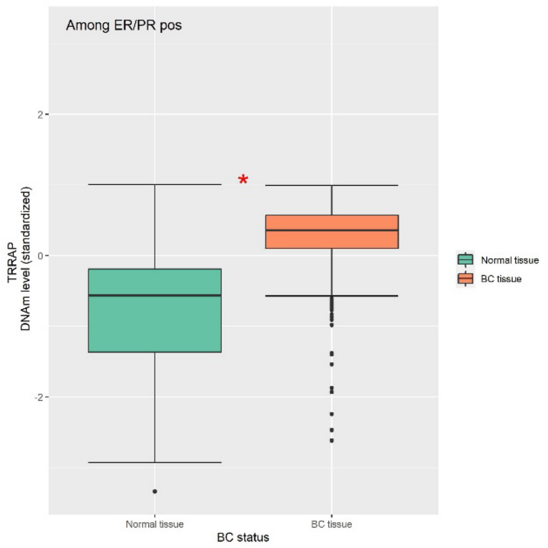

R. Among ER/PR pos: *CALHM1*

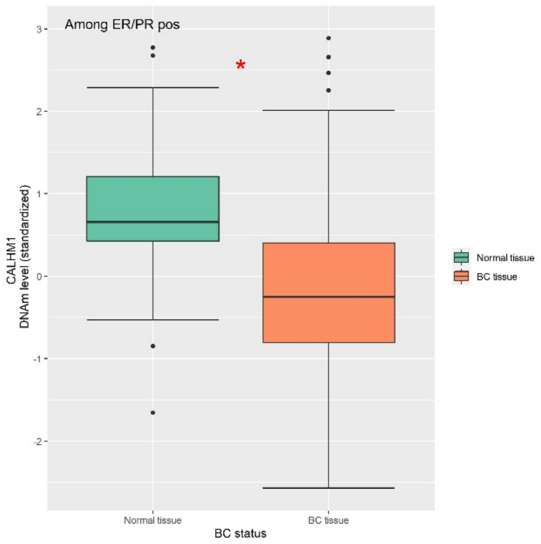

Figure S4. Box plots for DNAm levels from EWA IR-CpGs in association with BC, shared by non-BC datasets according to Chr, CpG context, and gene region. (BC, breast cancer; Chr, chromosome; CpG, CpG dinucleotide; DNAm, DNA methylation; EWA, epigenome-wide association; IR, insulin resistance; TCGA, The Cancer Genomic Atlas; UTR, untranslated region; WHI, Women's Health Initiative. * Statistical significance after multiple comparison correction.)

A. By Chr

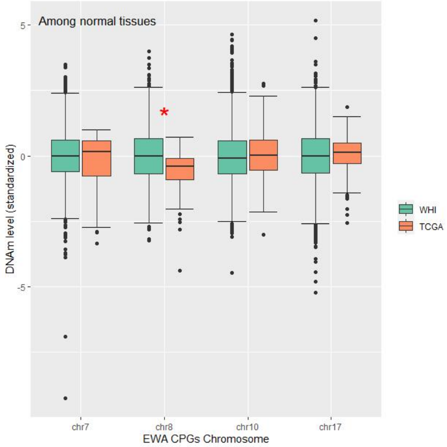

B. By gene region

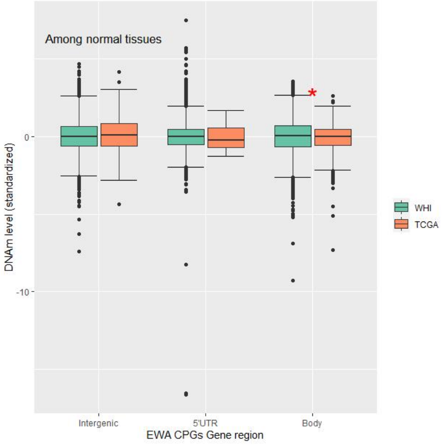

C. By CpG context

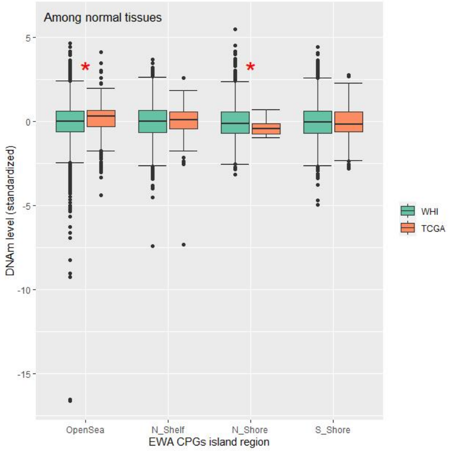

D. By enhancer

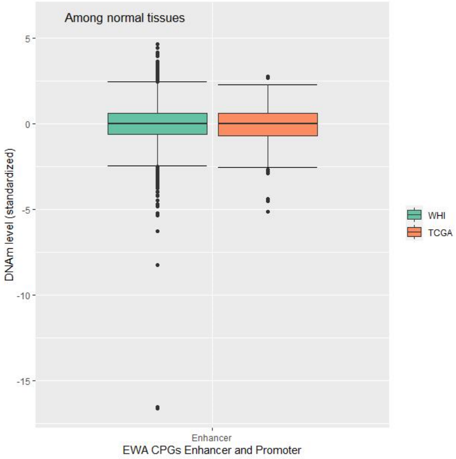

E. By 1 CpG (cg01676795)

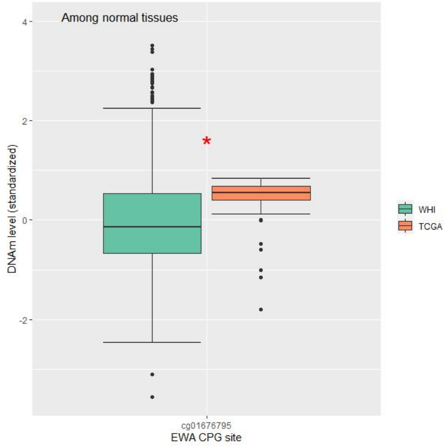

Supplement: Supplementary file 1 — Additional file 1: Additional tables and figures. [file 13148_2023_1435_MOESM1_ESM.pdf]
